# A fingerprint approach to pioneer structure-based T cell receptor repertoire analysis and specificity prediction

**DOI:** 10.3389/fimmu.2025.1688805

**Published:** 2025-11-07

**Authors:** Francesca Mayol-Rullan, Marine Bugnon, Marta A. S. Perez, Vincent Zoete

**Affiliations:** 1 Computer-aided Molecular Engineering Group, Department of Fundamental Oncology, Lausanne University, Ludwig Institute for Cancer Research, Epalinges, Switzerland; 2 Molecular Modelling Group, SIB Swiss Institute of Bioinformatics, Lausanne, Switzerland

**Keywords:** T cell receptor (TCR), antigen, specificity, fingerprints, 3D structure

## Abstract

**Introduction:**

The development of cancer immunotherapy has accelerated in recent years. Understanding the specificity of T cell receptors (TCR) for peptides presented by the major histocompatibility complex (pMHC) is a critical step towards improving immunotherapy approaches, such as adoptive cell transfer and peptide vaccination. Despite notable computational advances, the unambiguous pairing of TCR with pMHC, from pools of thousands of candidates and unseen pMHC, remains elusive.

**Methods:**

To meet this challenge and showcase the potential of using physics-based structure-based methods without being hindered by their computational cost, we developed a novel approach, TCRfp. This method transforms the 3D structure of TCRs into one-dimensional structural fingerprints (FPs) using the electroshape 5D (ES5D) technique.

**Results:**

We have modelled more than 15’000 3D structures of paired TCR alpha and beta chains with known sequences and pMHC specificity and encoded them into 1D TCRfp. Anticipating future clinical applications, we have translated the TCR modelling process into a fast pipeline. Similarity measures between TCR FPs correlate with their ability to recognize similar or identical epitopes within both the training set and in the external validation sets.

**Discussion:**

TCRfp constitutes a rapid approach for high-throughput TCR comparison and repertoire analysis based on molecular 3D structures. When tested on a private dataset and combined with a basic sequence-based method via logistic regression, TCRfp surpassed existing approaches in predicting TCR specificities. TCRfp represents a structurally informed complement to sequence-based approaches and could enhance our ability to decode immune recognition.

## Introduction

Cancer immunotherapy development has been in the rise over the last years. One of the most promising research topics in this field involves the understanding and unambiguous prediction of T-cell receptor (TCR) recognition of a specific cancer epitope (i.e., peptide-MHC, pMHC). Predicting the TCR-pMHC specific interactions is essential for improving immunotherapies such as adoptive cell therapy or peptide vaccination. Nevertheless, understanding the mechanisms underlying the TCR-pMHC specificity remains practically unresolved due to its complexity (1, 2).

Over the past years, TCR-pMHC specificity has been widely explored by several authors who developed numerous computational approaches (2, 3). Those that predict TCR-pMHC specificity can be divided into sequence-based and structure-based methodologies. Ongoing improvements in sequencing technology (4–7) are yielding more numerous and reliable TCR-pMHC sequence pairs (8–10), accelerating the development of better, more accurate in silico approaches. As an example, biotech companies such as Adaptive Biotechnologies in partnership Microsoft Healthcare NExT initiative (11) are providing extensive TCR data and TCR mapping. Still, the number of TCR-pMHC pairs available corresponds to a tiny fraction of the overall possibilities. Typically, sequence-based approaches use machine learning techniques such as logistic regressions and deep neural networks that use sequences to train a function that attempts to correctly predict if a TCR can bind a given epitope present in the training set (12–15). These models can achieve high performance for TCR classification, but they still require substantial sequence datasets to provide sufficient predictive power for their algorithms. Consequently, they show limited success in predicting TCR specificities for unseen epitopes or epitopes and alleles with very limited representation in the training data. Structure-based approaches provide a learning set-independent alternative. They use experimental structures or structural models of the TCR-pMHC complexes to determine the most likely ones based on estimates of the affinities between partners, using universal physics-based scoring functions (16–20). Despite great successes, structure-based approaches are time-consuming and hardly tractable for large scale predictions. The increase of the number of TCR-pMHC structures (230 TCR-pMHC class I and 82 TCR-pMHC class II as of 5^th^ of July 2023) in the Protein Data Bank (PDB) (21)together with the appearance of more powerful tools to create 3D models out of sequences such as TCRmodel (22), TCRmodel2 (23), AlphaFold (24) and LYRA (25) are contributing to the development of new structure-based approaches (19).

This paper introduces TCRfingerprint, TCRfp, a novel 3D-based method designed to address the specificity of TCRs in recognizing pMHC complexes. The method builds upon the ElectroShape approach (ES5D) (26, 27), which extends traditional **3D atomic representations** by incorporating two additional physicochemical dimensions: atomic charge and lipophilicity, resulting in a comprehensive 5D fingerprint. While previous studies have employed Kidera factors and other physicochemical features to predict TCR–epitope interactions (28–30), the use of the ES5D framework to encode de 3D shape and the spatial distribution of charge and lipophilicity in this context is entirely new and forms the core innovation of our method. This enables TCRfp to cluster TCRs in a way it correlates with their specificity, particularly when the specificity is driven by shape and physico-chemical characteristics rather than sequence similarity. Although building a TCR 3D model is the most time-consuming step in our pipeline, it takes only 1.4 minutes on a 16-CPU machine, and the subsequent conversion of the 3D structure into a fingerprint on the millisecond scale. Our approach is thus much faster than the usual purely 3D-based approaches which model the full putative TCR-pMHC complexes and estimate binding free energies. Additionally, unlike sequence-based approaches, TCRfp does not require to be retrained for each pMHC, enabling its application to unseen epitopes. TCRfp pioneers a new class of fast structure-based approaches for TCR analysis, clustering and potentially deorphanization. TCRfp demonstrates its value by offering supplementary insights when sequence-based methods fall short. When applied to a private test set and combined with a basic sequence-based approach through logistic regression, TCRfp surpassed existing approaches to predict TCR specificity.

## Materials and methods

### Data retrieval, cleaning and curation

**Figure 1:**
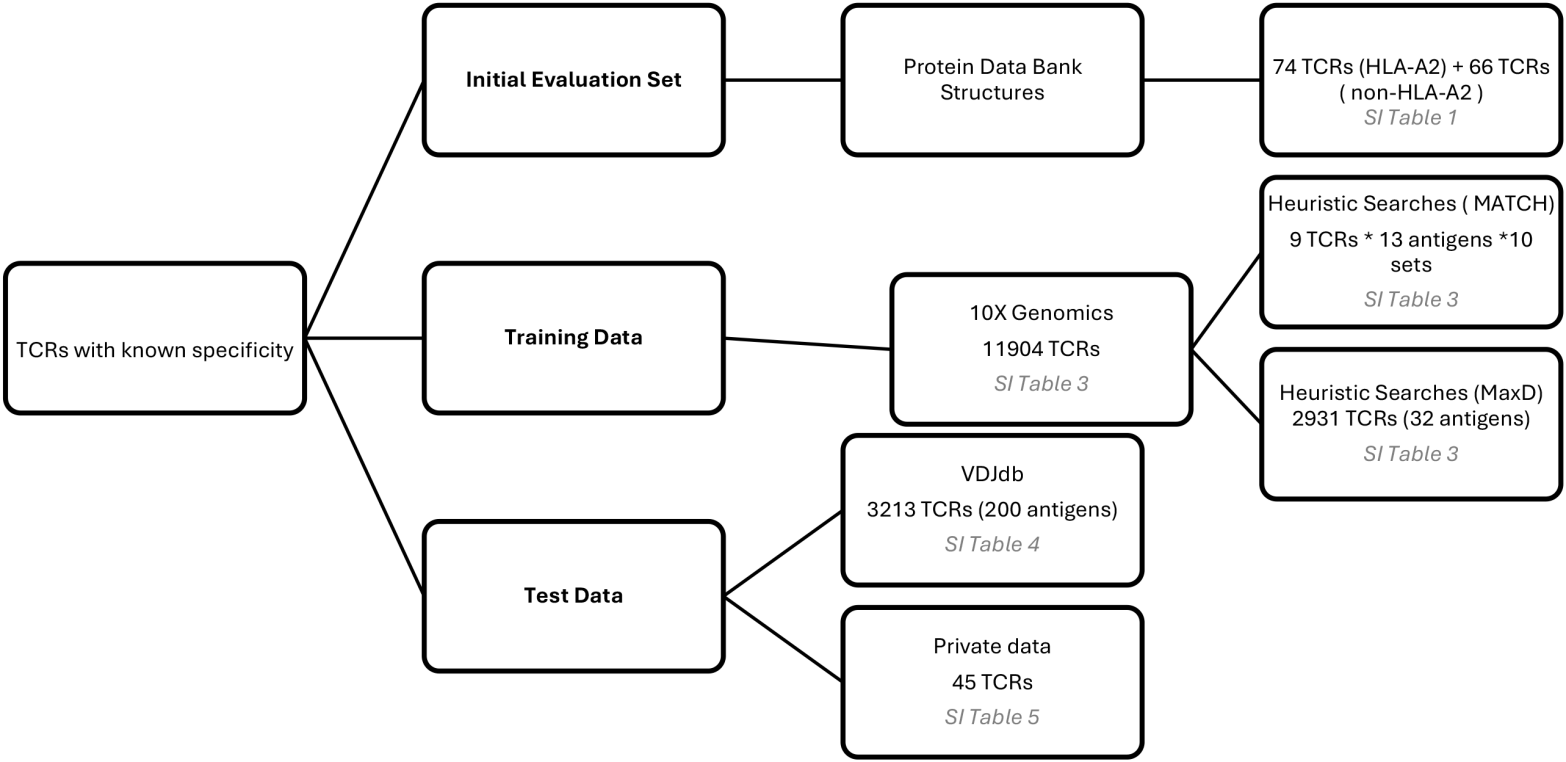
Datasets of TCRs with known pMHC specificity used during the development of the TCRfp: baseline set, training data and test data.

Multiple datasets of TCRs with known pMHC specificity were employed during the development of TCRfp. Initially, a baseline set, comprising 3D TCR-pMHC structures obtained from the Protein Data Bank (31), was used to evaluate the default method based on domain knowledge and informed assumptions. Subsequently, two independent training sets from 10x Genomics [CD8+ T cells from human Healthy Donors 1, 2, 3 and 4 (v1, Single Cell Immune Profiling Dataset by Cell Ranger 3.0.2, 10x Genomics, (2019, November 25))] were incorporated to perform heuristic parameter searches aimed at optimizing model performance. Finally, two external test sets, not included in any training or baseline sets, were used for evaluation: one derived from the VDJdb (32) and another from a private dataset. A summary of these datasets and their respective roles is presented in **Figure 1** with further details presented below in that section.

#### TCR structures with known pMHC from Protein Data Bank

To perform a preliminary analysis of our 3D-based approach, we used a set of 74 TCR structures with known pMHC (HLA-A2 restricted) taken from PDB as of September 2019. TCR-pMHC complexes were downloaded and then cleaned by removing pMHC, non-standard amino acids, water molecules and other TCR molecules in case several TCR dimers were present. In the end, we only retained the structure of one TCR pair (one α and one β chain). The PDB ID (PDBid) of the structures, the label of the retained chains, and the peptide (pMHC = pHLA-A2) can be seen in **Supplementary Table 1**. To calculate the sequence recapitulation (see definition below in this methods section), peptides’ sequences were aligned based on their structural superposition using UCSF Chimera (33). Kinematic closure (KIC) method (34) of the Rosetta software version 3.11 (35) was used to create 100 different low energy conformations of the 6 CDR loops of each HLA-A*02 restricted TCR. The lowest energy conformation per TCR determined using Rosetta REF15 scoring function (36) was selected as TCR model of the PDB structure for further analysis.

#### Development of a modelling pipeline based on Rosetta to create TCR models from sequences

To generate TCR 3D structures models from their sequences a fast and automated TCR homology modelling pipeline was developed. The pipeline starts by reading the sequence information from the TRAV, TRAJ, TRBV, TRBJ genes as well as the CDR3 of each TCR chain. Then, the CDR3 sequences are aligned with the TRV and TRJ genes from the respective chains, and the full α and β chain sequences are reconstructed. Subsequently, the TCR sequences are converted into 3D structures by homology modelling using Rosetta version 3.11 (35) and the TCR modelling protocol described in Gowthaman et al (22).

Benchmarking of the Rosetta modelling protocol using a set of nonredundant TCR experimental structures showed that models are accurate and compare favorably to models from other available modelling methods (22). Furthermore, in our own pipeline, 10 conformations per TCR were generated and ranked with the Rosetta REF15 scoring function (36) and the lowest-energy conformation was selected as the final TCR model. Finally, to detect and remove problematic models, all the final model conformations were subjected to distance-based quality controls. Indeed, by applying Rosetta protocol to model the TCR sequences from 10xGenomics we have observed that inaccurate TCRs models could be obtained (see results and discussion for further details). For this reason, we developed filters based on the distances among specific conserved residues within the TCR, using the distances found among the conserved residues from 92 experimental structures (**Supplementary Table 2**). To implement these filters, the TCRs were first renumbered according to IMGT (37) numbering using the ANARCI program (38) to keep the same residue number for constant residues across all the TCRs (38). Based on (39), 4 conserved residues were selected for the filters (CYS23, LEU89, CYS104, TRP118). The distances between 9 combinations among them were calculated with UCSF Chimera (40) using a set of 92 experimental structures of TCR class I (**Supplementary Table 2**). For a model to be accepted as accurate, the distances must lie within the range defined as the average value in the experimental structures ± 4 times their standard deviation for each filter. The models that did not match the distance filter criteria were discarded.

### Training and validation sets

#### Training sets

CD8+ T cell sequences with known cognate pMHC were retrieved from the 10xGenomics database on the 25^th^ of November 2019 (CD8+ T cells from human Healthy Donors 1, 2, 3 and 4 [v1, Single Cell Immune Profiling Dataset by Cell Ranger 3.0.2, 10x Genomics, (2019, November 25)]. The dataset was carefully cleaned by removing unreliable sequences. Additionally, all the retrieved T cell sequences lacking relevant information – i.e., a whole chain, a single gene, CDR3 data or the peptide specificity - were excluded. As the data used from 10xGenomics was obtained through Single-Cell Sequencing, we had to construct the paired TCRs. During this process we encountered a high proportion of TCRs showing abnormal sequence count: TCRs with multiple α, β or both chains. Biologically, a fraction of T cells is known to express multiple α and β chains: two α and one β or vice versa (41). To remove all possible ambiguities, we kept only TCR from T cells expressing a single α and a single β chain. After applying these stringent filters 14’479 TCR sequences with known pMHC were retained. TCR sequences were then converted into 3D structures by applying the protocol previously described. Our TCR data set consists of 11’904 TCR models from 9 MHC and covers specificities for 33 different peptides (all the detailed data can be found in the **Supplementary Table 3**). Surprisingly, the majority of the TCRs found within our dataset are specific for the KLGGALQAK peptide (73.7%), followed by GILGFVFTL (7.7%), AVFDRKSDAK (5.8%) and RAKFKQLL (5.6%). The rest of the antigens account for 2% or less of representation from the total dataset.

#### Creating TCR datasets to optimize TCRfp via heuristic search (MATCH)

To avoid over or under representation of a peptide in respect with the others we constructed 10 training sets. Each set was composed of groups of 9 TCRs, each group binding one of 13 different antigens, for a total of 117 TCRs per set (specified in the **Supplementary Table 3**).

#### Optimizing TCRfp by maximizing the distance between TCRs (MaxD)

To mitigate the overfitting that could be generated by small training sets, we examined optimizing the parameters of TCRfp to maximize the average distance between all TCRs considered. As this approach does not require an equal number of TCR binding the same peptide, we included all the TCR models data in a single dataset. Again, TCRs binding to several peptides were discarded beforehand to avoid biases in the scoring. However, it was possible to include peptides recognizing a single TCR, which were discarded in the previous MATCH training datasets, as the TCR specificity was not required in this new approach. To avoid a bias towards the peptide KLGGALQAK all the corresponding models were discarded from the training. The final training set consists of 2’931 TCRs that covered specificities for 32 different peptides (**Supplementary Table 3**).

#### External test set

To assess the performance of our FP we used a different set that does not include any of the previous TCRs used for the training. This external test set was retrieved from the VDJdb curated database (data obtained in 2022). We modelled the TCR 3D structures using our TCR modeling pipeline, obtaining a total of 10’703 TCR structures to use as an external validation dataset for our FP scoring. To avoid a bias towards our results, all the redundant TCRs overlapping with the 10xGenomics database were excluded. Additionally, we discarded the TCRs recognizing the overrepresented peptide KLGGALQAK (53.6%) and the singleton TCRs that are impossible to pair (and so, useless for our test exercises). The final validation set consists of 3’213 TCRs, covering specificities for 200 different antigens. All the TCRs structures used in the final validation, including their genes composition and peptide specificity, are described in **Supplementary Table 4**.

#### Test set of 45 private TCRs

An additional test set consisted in a private collection of 45 CD8+ TCR sequences with known pMHC (**Supplementary Table 5**) found in 4 melanoma cancer patients (Mel #1, Mel#2, Mel#3 and Mel#4). These TCRs did not overlap with the TCRs used in the developmental set and in the previously mentioned external test set.

### Adaptation of the ElectroShape algorithm for TCR models

We have applied ElectroShape 5 Dimensions (ES5D) (26, 27), to convert experimental and modelled TCR 3D structures into 1D vectors. The ES5D algorithm translates the 3D structure of the TCR in a 1D numerical vector, relying on 6 centroids. The latter are points of interest surrounding the molecule to describe (see latter for the definition of their positioning). In this approach, all centroids and atoms have 5D coordinates: the 3 Cartesian coordinates, the weighted atomic partial charges (4^th^ dimension) and the weighted lipophilicity (5^th^ dimension). The latter is defined as the atomic contribution to logP according to the WLOGP algorithm (42). Default weights for the atomic partial charges and lipophilicity were established at 25 and 4, respectively. These values found to be a good couple for small molecules were also effective for discriminating TCRs. To obtain the final 1D vector that defines the structural FP of a TCR, the average, standard deviation and third moment of the distances between all atoms (including hydrogen atoms) of the TCR’s CDR loops to each of the centroids were calculated, leading to a final vector of 18 values.

Once a FP has been calculated for each TCR, quantifying the similarity between two TCRs using ES5D boils down to calculating the distance between a pair of 1D vectors with a Manhattan distance-based score, which ranges from 0 for TCRs with totally dissimilar shapes to 1 for TCRs with perfectly identical shapes:


$$\text{Similarity-score }={\left(1+\frac{1}{n}\sum_{1\leq i\leq n}\left|x_{i}^{TCR1}-x_{i}^{TCR2}\right|\right)}^{-1}$$

where *n* is the number of entries in the 1D vectors and *x* are the entry values of the vectors for each TCR.

### TCR similarity scoring

To assess that our FP-based Similarity-score correlates with the likeliness of TCRs to share the same specificity we have developed two other scoring systems based on the recognized peptide: the *sequence recapitulation* and the *peptide identity*.

The *sequence recapitulation* score estimates for each TCR within a set of TCRs with known specificity, if the closest TCRs among this set recognize peptide sequences with high degree of similarity. This is calculated by aligning the peptide sequence recognized by each TCR, taken as a reference, with the one of the closest TCR in the set according to our approach (excluding the TCR reference itself), and counting the number of identical residues in the alignment. The average value across all TCRs in the set represents the *sequence recapitulation* score. This score is particularly well adapted to test the approach on TCR-pMHC experimental structures, where several TCRs among the set are binding peptides baring a few point mutations.

The *peptide identity* score measures, for each TCR in each set, how frequently the closest TCR according to TCRfp binds the same peptide. This score is relevant when studying datasets with TCRs that recognize only a few unrelated peptides. Using a set of TCRs for which the cognate pMHC are known, the process involves identifying if they bind the same peptide (positive pair) or a different peptide (negative pair). The fraction of positive pairs among all pairs defines the peptide identity score expressed in %.

### Centroids definition

To translate TCR 3D structures into 1D fingerprint vectors, we initially placed each of the 6 centroids on the α carbons of the tip of the loops of one of the 6 CDRs (CDRs 1, 2, 3 from α chain and CDRs 1, 2, 3 from β chain). In the case of loops with an even sequence length, the tip of the loop was selected by taking the lowest from the two middle residues. As the tip of the loops are situated in flexible TCR regions and contact the pMHC, these represent strategic places to better describe the 5D shape of the TCRs. As mentioned earlier, the 4^th^ and 5^th^ dimensions of the atomic coordinates are defined as the weighted atomic partial charge and the weighted lipophilicity, respectively. To perform our first tests, after exploring a limited number of combinations (such as 25;4, 35;4 and 45;4), the charge and lipophilicity weighting parameters (C and P), were initially set to 25 and 4, respectively, since this combination was shown to perform the best. The C and P combination of values that were tested and finally used were inspired from previous studies with ES5D and small molecules (26) and a study performed in the lab (data not shown). These C and P values were later optimized by heuristic search, as described below.

### Genetic algorithms with *peptide identity* scoring

We used genetic algorithms (GAs) to investigate the possibility of defining universal centroids in 5D space that could effectively represent all TCRs. Given the huge sample space, (6 centroids each positioned in 5 dimensions plus C and P weighting parameters to explore), GAs were well-suited for this optimization. To enable the identification and use of such universal centroids, we first superimposed all the TCRs by centering them at the origin of the 3D space and aligning their main axes along the axes of the Cartesian space using the COOR ALIGN function of the CHARMM program.

Our GA performs as follows:

1. Initialization of the population: the optimizable solution, which characterizes each individual of the population, was defined as the 5D coordinates of the six centroids, together with the C and P weighting parameters, for a total of 32 values to be optimized. The initial highly diverse population consisted in 400 individuals with a different solution chosen at random.
2. Fitness calculation: The fitness of each solution, was calculated as the average *peptide identity score* over the 10 sets of 117 TCRs, as defined above. Briefly, each solution was individually applied to each set, and a FP was generated for each TCR of the set. Thus, for each solution under consideration we obtained different TCR FPs. Solutions improving the overall TCRfp efficacy was characterized by increased values of the average *peptide identity score*.
3. Parent selection: The 50% best solutions (200), i.e., those showing the highest *peptide identity score*, were selected for the reproduction step to produce the next generation.
4. Crossover and/or mutation: The next generation offspring was built by applying mutations and/or crossovers to the solutions of the selected parents. For the crossover, a random number of centroids – between 1 and 6 - were exchanged between the selected parents (depending on the variant of algorithm used, the centroid can be entirely exchanged or just partly). The weighting parameters C and P could also be exchanged individually at random. From the two possible children generated this way, only one was randomly selected. A random number, between 2 and 4 (other values were explored but those were kept based on their higher performance), of solution entries were selected and randomly incremented by a random value ranging from -1 to 1. 200 children were generated this way before entering the population. Finally, the *peptide identity score* was estimated for each individual and the 200 ones with the worst scores were discarded to keep a population of 200 individuals. The GA was stopped when the fitness score of the best individual had not improved for at least 20 generations. The overview of the genetic algorithm pipeline can be visualized in the **Supplementary Figure 2**.

Due to the large number of parameters to tune, finding the global optimum via this exploration would be considerably time consuming. We intended to perform a profound exploration of the best combinations of parameters to speed-up the search and to find near-optimal centroid values. This search was performed over 990 independent runs with different parameter combinations. In some runs, the centroids in the solutions were initially placed on the tip of the loop of each CDR (as specified for the original definition) and optimized starting from that position. To give more freedom to the algorithm to explore the search space, we also tested in some runs to initiate the centroid coordinates from random positions in the 3D space within a defined area of 30x30x30 Å^3^ (centered in the average middle of all the superimposed TCRs) and allowing them to move freely outside and inside that space. Summaries of the GA best algorithms can be seen in **Supporting Information Table 6** and the detailed GA parameters that led to best genomes can be seen in **Supporting Information Table 7**.

### Genetic algorithms with *FP distance* (MaxD) scoring

The GA was modified by introducing another scoring system for the selection criteria. Instead of using the *peptide identity score*, the GA fitness was defined as the highest distance among all the FPs of the TCRs of the set that do not recognize the same pMHC. The GA objective was then to maximize this fitness. The principle of this fitness was to separate as much as possible the TCR that do not bind the same peptide, while others could remain close to each other. Unlike the *peptide identity score*, this new fitness did not require to match TCRs according to their specificity. As the results were not biased by the proportion of TCRs per antigen, a different training set was used. The latter was composed of 2’931 TCR models that account for the specificity of 32 antigens. All the TCRs binding to the peptide KLGGALQAK were excluded from the pool of TCRs used by the GA to avoid an overrepresentation of this peptide in the results. Summaries of the GA best algorithms can be seen in **Supporting Information Table 6** and the detailed GA parameters that led to best genomes can be seen in **Supporting Information Table 7**.

### Comparison of TCRfp with a sequence-based approach

Most of the approaches analyzing TCR repertoires and TCR specificities are based solely on their sequence. Contrarily to them, our approach encodes information regarding the biophysical and structural characteristics of the TCRs. To compare the performance of TCRfp with those of sequence-based approaches, we applied a sequence-based approach on the same validation set used for the validation of the TCRfp. BLOSUM matrices are usually used in sequence alignment algorithms to assess the similarity between sequence alignments. For this task, we calculated the BLOSUM62 score to assess the sequence similarity among the TCR sequences with an open gap penalty of -3 and an extension gap penalty of -1 for each TCR compared to the rest of TCRs of the validation set (43). Of note, the scores given by this BLOSUM62 calculation can reach negative values. Hence, we normalized the BLOSUM score values so it would range from 0 to totally different TCR sequences to 1 for identical TCR sequences, like TCRfp. Using the *BLOSUM62 score*, it was possible to calculate *peptide identity scores* and compare with those obtained using TCRfp.

### Logistic regression that combines the sequence-based score with TCRfp

We have implemented a logistic regression, based on the FP and on the *BLOSUM62 score*, to determine the probability of a TCR pair to bind the same peptide. The probability, *p*, of a TCR pair to recognize the same peptide was given by:

The bias term b0 and the weights Wn were determined using a set of 3213 TCRs (validation set, **Supplementary Table 9**), maximizing the likelihood that each TCR pair shares the same specificity. The accuracy of the logistic regression was determined by the area under the ROC curve (AUC) and by the % of correct pairs. The best model had a bias term b0 equals to -4.4545 while the weights W1 and W2 were 7.3739 and 0.5752, respectively. The AUC was only 0.697, emphasizing the difficulty to discriminate between TCRs that share the same specificity and TCRs that do not share the same specificity. The threshold of the classifier was set to 0.5, and we predicted TCRs sharing the same specificity if P>0.5. The regression trained on the 3213 TCRs was then applied to a private set of TCRs showing that the Logistic Regression is better than sequence-based score and or the FP alone.

### Comparison of TCRfp with competing approaches

We used the private set of 45 TCRs from 4 melanoma patients (**Supporting Information Table 5**) to compare TCRfp with other approaches. This ensured a fair comparison, as this set of TCRs with known specificities was not used by us or by our competitors to develop the approaches.

On top of comparing TCRfp with a purely sequence-based approach and with a logistic regression that combines TCRfp and the sequence-based we have also compared with the gold standard approaches TCRpcDist (30), TCRdist3 (44) and TCRbase (web server: https://services.healthtech.dtu.dk/services/TCRbase-1.0/).

Each single TCR in the private set of 45 TCRs was compared with the other 44 TCRs. We analyzed how often the nearest neighbor of a given TCR in the private set shared the same specificity.

For TCRbase, when comparing a given TCR with the other 44 TCRs, the web service only provided the query TCR and its nearest neighbor as output. With this, to find the two closest TCRs for a given TCR within our private set (rank 2), we had to:

1. Input the test TCR and screen it against 44 TCRs (reference set).
2. Wait and retrieve the nearest neighbor from the output.
3. Input the test TCR again and screen it against 43 TCRs (excluding the previously obtained nearest neighbor) to get the second nearest neighbor.

This iterative process complicated direct benchmarking and extended analyses. We are providing TCRbase results for rank1 and rank2.

### Receiver operating characteristic curves and other metrics for statistical analysis

To test the predictive power of TCRfp, we defined a TCR classifier that assigns a given TCR to the TCRs within the repertoire with the highest similarity. In other words, for a given TCR, the closest TCR is the TCR with the highest TCRfp similarity among all the TCRs within the dataset, excluding itself. We measured the sensitivity and specificity of the classifier. To test the predictive power for a given pMHC, we did 5 cross-validations by randomly allocating for a given data set 70% - 30% as a training and test sets, respectively. We tested how often the TCRs with the highest similarity/similarities recognize the same peptide when compared with the reference at different ranks and thresholds. We also tested it by measuring sensitivity and specificity. For each given pMHC, the average of the area under these receiver operating characteristic curves (AUC), a standard metric of the classification success, was calculated together with its standard deviation.

## Results

### TCR fingerprint approach, TCRfp

**Figure 2:**
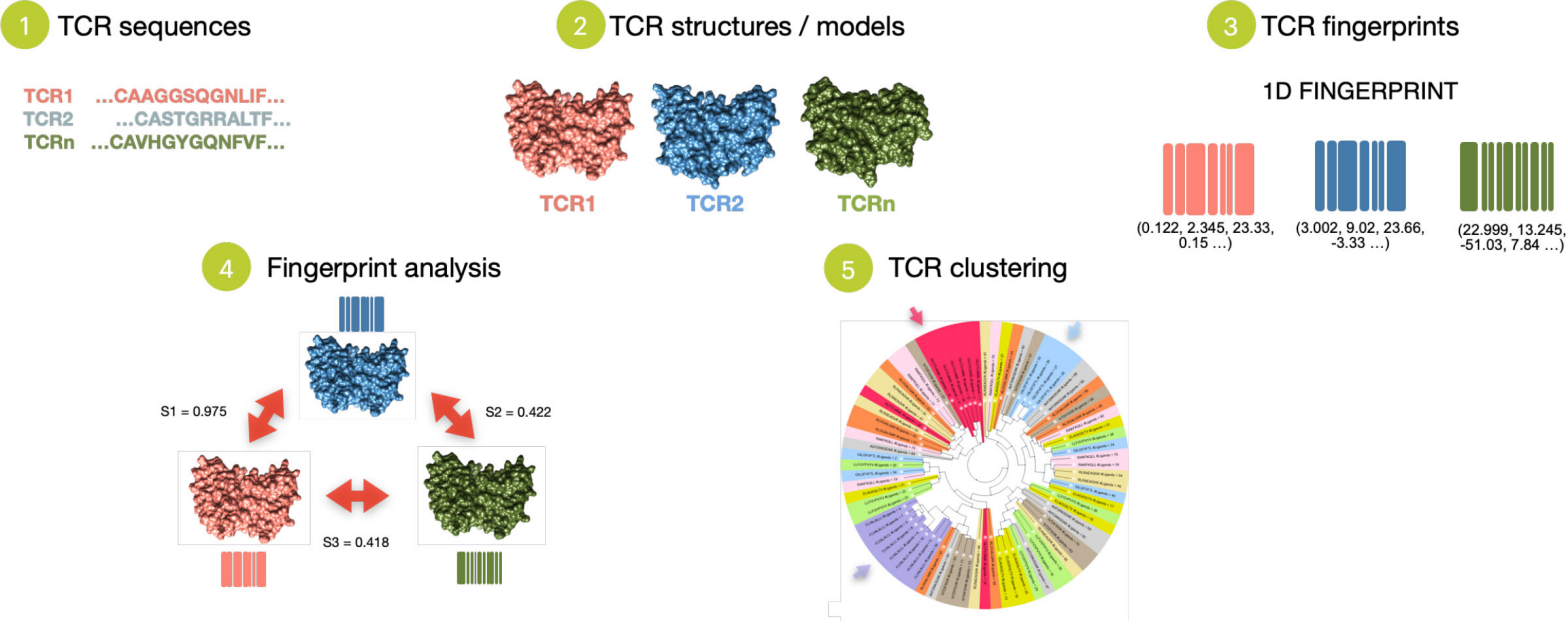
Schematic overview of the TCRfp approach. TCRfp is described in the main first three steps: 1. TCR sequences are retrieved and 2. converted into structures using homology modelling to be further 3. translated into a 1D numerical representation known as the FPs. With this vectorial representation, the TCR structures can be easily compared through the FPs (4) and even clustered (5) using different methods such as UPGMA hierarchical clustering.

The T-Cell Receptors fingerprint approach, TCRfp, consists in 5 main steps. TCR sequences are first retrieved and secondly converted into 3D structures using TCRmodel (22, 45). Third, the 3D structure of each TCR is converted into a simple fixed-length numerical representation, the fingerprint (FP), using a variant of the ES5D algorithm (26, 27) specifically adapted to TCR structures. This modified ES5D algorithm translates the complementary determining regions (CDRs) of the 3D structure of the TCR in a 1D numerical vector, relying on 6 centroids – spatial references to calculate the fingerprints and described in detail below. In this approach, all centroids and atoms have 5D coordinates: the 3 cartesian coordinates, the weighted atomic partial charges (4^th^ dimension) and the weighted lipophilicity (5^th^ dimension). The 5^th^ dimension is derived from the atomic contribution to logP using the WLOGP algorithm (42). Default weighting parameters for the atomic partial charges and lipophilicity were set to 25 and 4, respectively. These values which are well suited for small molecules also prove to be effective for discriminating TCRs. To calculate the final 1D vector representing the TCR’s FP, the average, standard deviation, and third moment of the distances between all atoms (including hydrogen atoms) in the TCR’s CDR loops and each centroid are computed, resulting in a vector of 18 values (3 values per centroid). Fourth, TCR comparison is performed via the calculation of Manhattan distance between 1D-vectors, with similarity (similarity score) ranging from 0 for TCRs with totally dissimilar shapes to 1 for TCRs with perfectly identical shapes. TCRfp is based on the similarity principle (46) according to which two structurally similar TCRs - that share close FPs - are more likely to bind to the same or to a similar pMHC. Fifth, TCR repertoires are analyzed, for example, by clustering TCRs based on the similarities between their FPs (**Figure 2**).

### Development of a pipeline to obtain accurate TCR 3D structural models from their sequences

**Figure 3:**
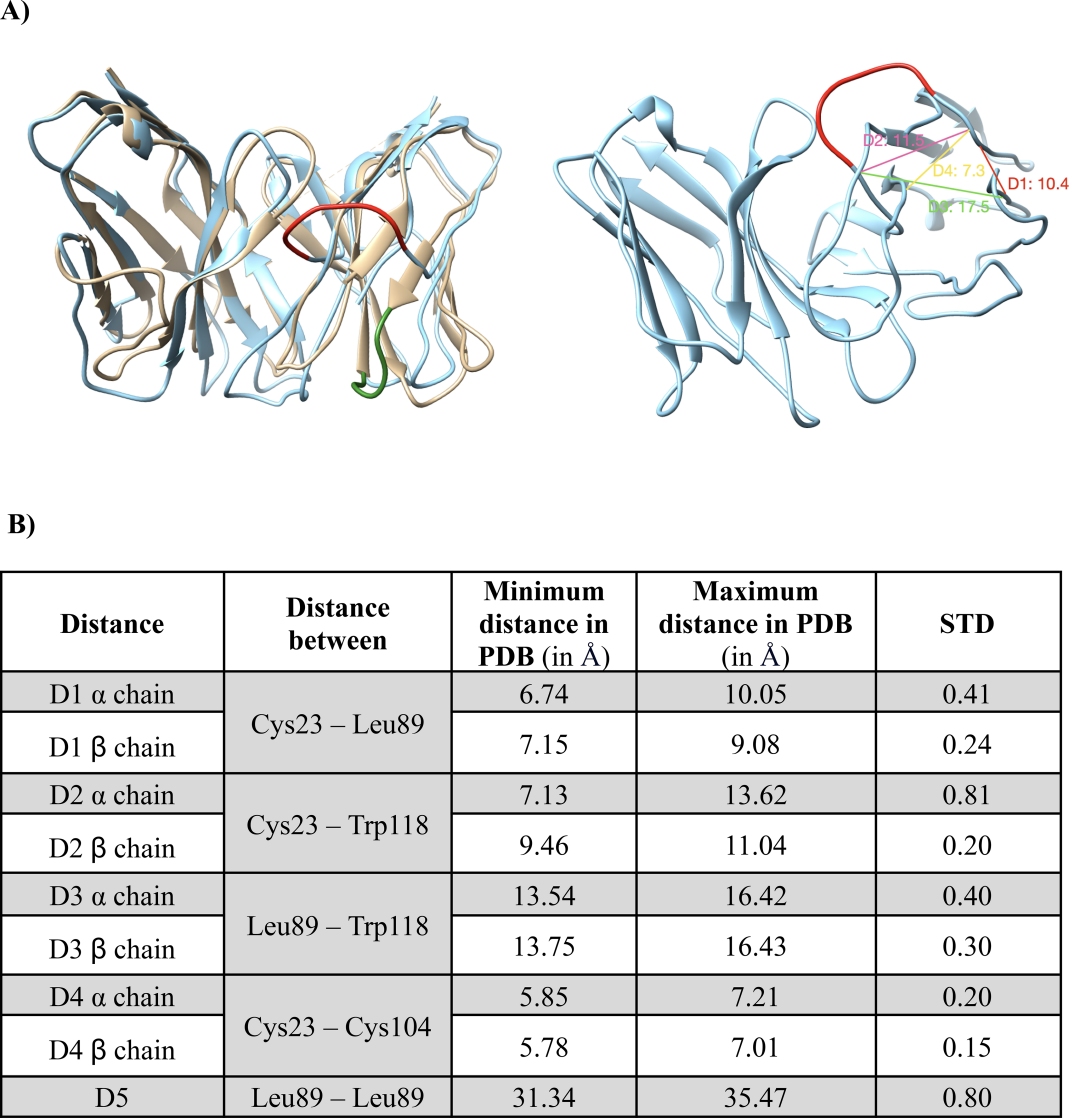
Distance-based validation filters for the TCR modeling. **(A)***on the left side*: Superimposition of a wrong TCR model (10X ID 10219: TRAV19, TRAJ24, CDR3α: CALSEADDSWGKLQF, TRBV27, TRBJ2-2, CDR3β: CASSLYGNLGTGELFF, binding the peptide KLGGALQAK) with a good TCR model (10X ID 1627: TRAV38-2DV8, TRAJ48, CDR3α: CAYRAPRGNEKLTF, TRBV2, TRBJ1-1, CDR3β: CASSDRMNTEAFF, binding the peptide AVFDRKSDAK), colored in light blue and sand, respectively. A fraction of the β chain badly modelled is colored in red and for comparison, the corresponding structural element of the correct model is displayed and colored in green. *On the right side*: Th model exceeded the distance thresholds for D1, D2, D3 and D4 of the β chain. Each distance is shown in the figure with a different color. The red representation corresponds to the same one shown in the left figure for a better visual comparison. **(B)** Table detailing the distance ranges (in Å) and standard deviations (STD) used for the distance-based filters, calculated based on 92 experimental TCR structures. Accepted ranges of distances used to define satisfactory models are equal to the mean ± 4 standard deviations.

To model TCRs 3D structures from sequences we used the TCRmodel approach, whose ability to predict TCR structures was proved in its seminal paper (22). Addressing the inherent difficulty of loop modelling, while modelling thousands of TCRs from 10X Genomics dataset (10X), we pinpointed erroneous TCR models as demonstrated in the example provided in **Figure 3A**. This example corresponds to TCR ID 10219 (TRAV19, TRAJ24, CDR3α: CALSEADDSWGKLQF, TRBV27, TRBJ2-2, CDR3 b: CASSLYGNLGTGELFF). All TCRs modelled and their ID can be found in the **Supplementary Table 3**. To automatically detect these problematic models, we developed distance-based filters. For this, we selected specific non-variable residues, measured distances between their C*α* atoms (**Figure 3B**) and checked if they fall within acceptable ranges. The distance ranges were determined using 92 experimental TCR class I structures available in the Protein Data Bank (PDB) (47) (**Supplementary Table 2**) and were calculated as the mean ± four standard deviations (STD) for each one of the five distances considered (**Figure 3B**). If at least one of the distances fell outside the acceptable ranges, the corresponding TCR model was considered erroneous and was therefore discarded. Since TCRmodel can provide different loop models when repeatedly applied to the same sequence, we decided to conduct several attempts to produce 3D models falling within the predetermined acceptable ranges.

In brief, our modelling pipeline creates TCR sequences of both α and β chains using the TRAV, TRAJ, TRBV and TRBJ genes, as well as the CDR3α and CDR3β sequences as input. All the gene information and all the 10X TCRs successfully modeled with our pipeline are given in the **Supplementary Table 3**, including the information regarding the specificity. 10 attempts of conformations per TCR were constructed by homology modeling with the Rosetta TCRmodel approach and for each attempt a quality check was performed with distance-based filters, that could be applied thanks to the renumbering of the residues using ANARCI and the IMGT scheme that provided the same numbers to conserved residues. Only the attempted models that passed the quality checks were further considered and the lowest energy one according to Rosetta REF15 scoring function (36) was used to calculate the TCRfp. If after 10 attempts, none of the models for a given TCR pass the quality check, we assumed that it was not possible to obtain a reliable model and the TCR sequence was excluded. The accuracy of the modelling pipeline was tested within a set of 187 TCRs with known experimental structures, targeting class I and class II pMHCs. Importantly, to model each TCR from the set, the experimental structures sharing the same genes and that of this TCR itself were removed from the list of possible templates. This intentional exclusion to make the benchmark modelling exercise very challenging and closer to the typical real application. Comparing the models and the experimental structures of these TCRs shows that our pipeline could predict TCRs structures with a satisfying accuracy, illustrated by a global RMSD (on the heavy atoms and all CDRs) of 2.1 Å between models and experimental structures. However, 37 TCRs out of 187 could not be modeled due to a lack of relevant templates and 41 did not pass the stringent-based filters, totalizing 78 structures that were discarded. We explored the possibility of increasing TCRmodel accuracy, especially for CDR3s, by exploring different options for loop refinement (data not shown). However, none of the options achieved a better average accuracy, while computation time increased substantially. Consequently, these options were not applied to the final pipeline. Our modelling pipeline takes on average 1.40 minutes to model a TCR and can be distributed upon multiple cores, allowing 6’171 TCRs to be modelled in ~9 hours on a 16-cores machine. Using our modelling pipeline, 10’703 TCRs models were constructed for the 14’479 sequences in the 10X database.

### TCRfp – FP definition and initial assessment

The ES5D-based FP approach was adapted to TCRs from the initial small drug-like molecules approach by changing the definition of the so-called centroids, i.e. the spatial references used to calculate the fingerprints. Only the fraction of the TCRs 3D structures corresponding to the complementary determining regions (CDRs) of the TCRs, which constitute the most variable part of the receptor and are responsible for pMHC binding, were considered. Then, the respective FPs were calculated using 6 centroids, each one placed on the Cα of the middle residue of the 6 CDR loops (**Supplementary Figure 1**). The charge and lipophilicity of the Cα were used to compute the weighted charge and the weighted lipophilicity as 4^th^ and 5^th^ dimension for the centroids. The default weighting values of 25 and 4 were applied to the charge and lipophilicity, respectively. As a proof of concept, TCRfp was tested on a set of 74 3D-structures of TCR-pMHC (HLA-A*02) complexes from the PDB (**Supporting Information Table 1**). For each given TCR_ref_ (reference TCR) in this set, we identified the TCR with the highest Manhattan-based similarity according to their fingerprints (excluding TCR_ref_ itself) and compared the sequences of the peptides they recognize. We obtained an average sequence recapitulation of 76%, suggesting that the similarity calculated with this definition of the TCRfp strongly correlates with the pMHC these TCRs recognize. The *sequence recapitulation* score estimates for each TCR within a set of TCRs with known specificity, if the closest TCRs among this set recognize peptide sequences with high degree of similarity (Materials and Methods, subsection TCR similarity scoring). Interestingly, when a random TCR was chosen instead of the closest according to TCRfp, the average sequence recapitulation dropped to 32%, showing that our approach is much better than random (p-value< 0.0001). Of note, 28 TCRs are singletons (no other TCR in the set binds the same epitope). Removing them led to an average sequence recapitulation of 92%. Next, we extended the test by adding 66 non-HLA-A*02 restricted TCRs, totalizing 140 TCRs. For this larger and more challenging set, we found that the closest TCR to a given TCR_ref_ according to TCRfp (excluding TCR_ref_ itself), was binding the same pMHC in 64% of the cases. This is significantly more than the average sequence recapitulation that could be obtained by random picking (12%, p-value<0.0001). The average sequence recapitulation for this set, after removing 40 singletons, is 84%, showing once again that TCRfp strongly correlates with the pMHC these TCRs recognize.

Given the inherent flexibility of the CDR loops in TCRs, and recognizing that, in general applications, only TCR 3D models will be accessible rather than X-ray structures, we reassessed the average sequence recapitulation among TCRs post-remodeling of the CDR loops. This exercise is important to assess if the results obtained by TCRfp are sensitive to the imperfections inherent to structural models. Since only CDR residues are encoded in the TCRfp, this exercise could prove extremely challenging for our approach. Strikingly, the sequence recapitulation was maintained at 67% on the 74 individual HLA-A*02 restricted TCRs considered. This showed that X-ray structures can be replaced by structural homology models in our approach at a cost of a small reduction (9%) in the accuracy. These results proved that the similarity between TCR FPs calculated using this version of TCRfp strongly correlates with the pMHC they recognize, allowing to cluster TCRs showing the same pMHC specificity when TCR structures are not available.

### Explorations of alternative definitions of FP through heuristic searches

In the previous definition of TCRfp, TCRfp^TOL^, the centroids are placed on the Cα of the tip of the loop and are dependent on the TCR under investigation. This idea was inspired by the fact that the CDR loops are the regions determining the pMHC specificity and therefore the tip of the loop may serve as an optimal strategic location for the centroids. Defining centroids as structural elements of the molecules under study has the advantage of making it possible to calculate the FP for these compounds without having to first superimpose them all, which can be challenging for various molecules. However, since all the TCRs share the same common global 3D structure, notably for the constant part, TCR superimposition is straightforward to perform and we therefore decided to explore the possibility of using universal centroid positions, where the same 6 centroids defined by constant 5D, can be used to describe all the TCRs. As there is an infinite number of combinations of 6 centroids in 5D, we have explored them systematically, making use of heuristic searches based on genetic algorithms and two different objective functions, MATCH and MaxD (see Methods and **Supporting Information** labeled **DataSheet 2**).

### TCRfp definition using the tip of the loop shows the best performance

When applied to an external validation set of 3’213 TCRs, the definition of TCRfp, with centroids positioned on the tips of the loop, TCRfp^TOL^ provided an averaged *peptide identity score* of 29.8% (Rank 1; no threshold, p-value<0.0001 when compared with random). The best solutions of the heuristic searches, MATCH and MaxD, TCRfp^MATCH^ and TCRfp^MaxD^ runs led to averaged *peptide identity scores* of 28.2% and 29.6%, respectively (Rank 1; no threshold, p-value<0.0001 when compared with random 4.8% Rank 1; no threshold, p-value<0.0001). To assess the ability of our approach to pair TCRs with the same specificity at different FP similarity values, we calculated how frequently the TCR with closest distance (Rank 1) the two closest TCRs (Rank 2) and the 5 closest TCRs (Rank 5) share the same specificity (predictive ability). When considering a similarity threshold of 0.8 and rank5, TCRfp^TOL^ achieves a predictive ability of 75.48%, substantially better than TCRfp^MATCH^ (54.1%) and TCRfp^MaxD^ (36.5%). The extensive tunning process of the heuristic searches were outperformed by TCRfpTOL, whose parameters were not tuned on a training set. TCRfpTOL showed better predictive ability on an external validation set, produced more variable and distinguishable fingerprints, and avoided the complexity and overfitting of the heuristic searches. See **Supporting Information** labeled **DataSheet 2**.

### TCRfp ability to predict TCR specificity

**Figure 4:**
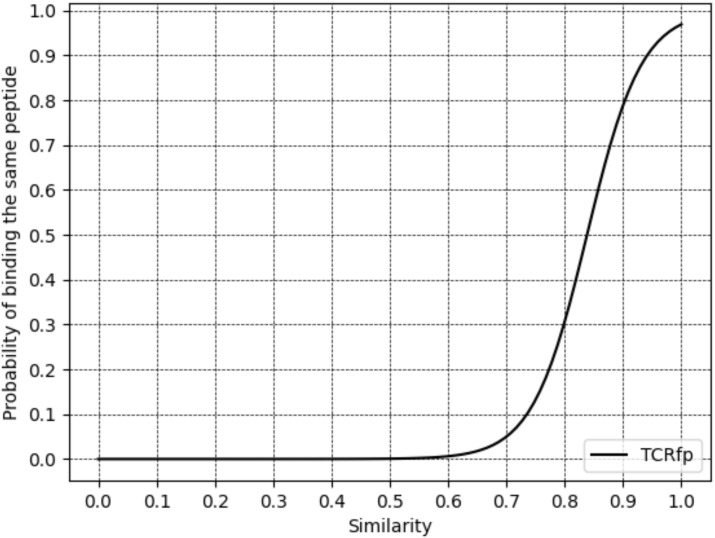
Relationship between TCR similarity (calculated for an external validation set of 3213 TCRs) and the probability of binding the same peptide at rank1.

To assess if the similarity score given by TCRfp can cluster TCRs in a way that correlates with their peptide specificity, we calculated the relationship between the TCRfp similarity between pairs of TCRs and the probability of these two TCRs to bind the same peptide (**Figure 4**). The similarity score ranges from 0 for TCRs with totally dissimilar shapes to 1 for TCRs with perfectly identical shapes. Interestingly, we find a clear sigmoid-like relationship between the similarity calculated by our approach and the probability of binding the same peptide. This relationship is very similar to the one found in the context of small drug-like molecules (48, 49), and supports the use of ES5D in the context of TCR repertoire analysis and specificity prediction. At a similarity threshold of 0.7, the probability of pairs of TCRs to bind the same epitope was 5%, increasing to 78.9% at 0.9 similarity, demonstrating TCRfp’s potential for high-precision epitope prediction.

Increasing the similarity threshold leads to a decrease in the number of TCRs that could be analyzed (**Table 1**). For example, at a similarity threshold of 0.8, only 26.0% of the TCRs could be clustered using TCRfp. This trade-off between prediction accuracy and the number of TCRs processed, as shown in **Table 1**, is also observed in other TCR-clustering algorithms (12, 50), which achieved 94% accuracy in predicting TCR specificity but only for 12% of the TCRs. The TCRfp demonstrated similar efficacy at the 0.9 threshold, with 95.0% accuracy for 12.3% of the dataset (**Table 1**).

**Table 1:** Accuracy pairing TCRs with the same specificity using TCRfp and a purely sequence-based approach (blosum-based) developed in house (see methods).

Approach	Threshold	Rank 1	Paired TCRs	Rank 2	Paired TCRs	Rank 5	Paired TCRs	Total TCRs	TCRs clustered
TCRfp	No threshold	29.8	956	34.8	1117	43.6	1382	3213	100%
	>0.7	35.9	898	40.6	1015	40.6	1199	2502	77.9%
	>0.8	75.5	631	78.5	656	78.5	683	836	26.0%
	>0.9	95.0	276	97.2	385	97.2	387	396	12.3%
Sequence-based	No threshold	41.4	1329	46.7	1502	54.4	1749	3213	100%
	>0.7	80.4	909	83.4	943	86.9	983	1131	35.2%
	>0.8	95.2	279	97.4	286	98.3	288	293	9.12%
	>0.9	100	11	100	11	100	11	11	0.34%

Accuracies calculated for different ranks and different similarity thresholds using a validation set of 3213 TCRs.

We focus on predictive ability based on ranks and thresholds as this closely mirrors the approach we plan to use in real-world applications. For instance, when assessing the specificity of an orphan TCR, we will compare it against a list of TCRs with known specificity, identifying the closest matches. Only the most similar (top-ranked) TCRs with high similarity will be considered. Using thresholds and rank-based analyses, Perez et al., employing an alternative approach, TCRpcDist (30), successfully predicted the specificities of orphan tumor-infiltrating lymphocytes in cancer patients, since the higher the similarity the greater the confidence in the predictions, as demonstrated in **Figure 4**. One might choose to analyze the full repertoire, accepting a lower overall accuracy, or instead focus on a smaller subset of high-confidence TCRs, thereby improving accuracy at the cost of losing coverage. Stringent thresholds can help prioritize strong candidates. Conversely, when such high-confidence matches are not found, relaxing the thresholds may be necessary, even if it slightly reduces predictive accuracy.

### FPs can complement sequence-based approaches

**Figure 5:**
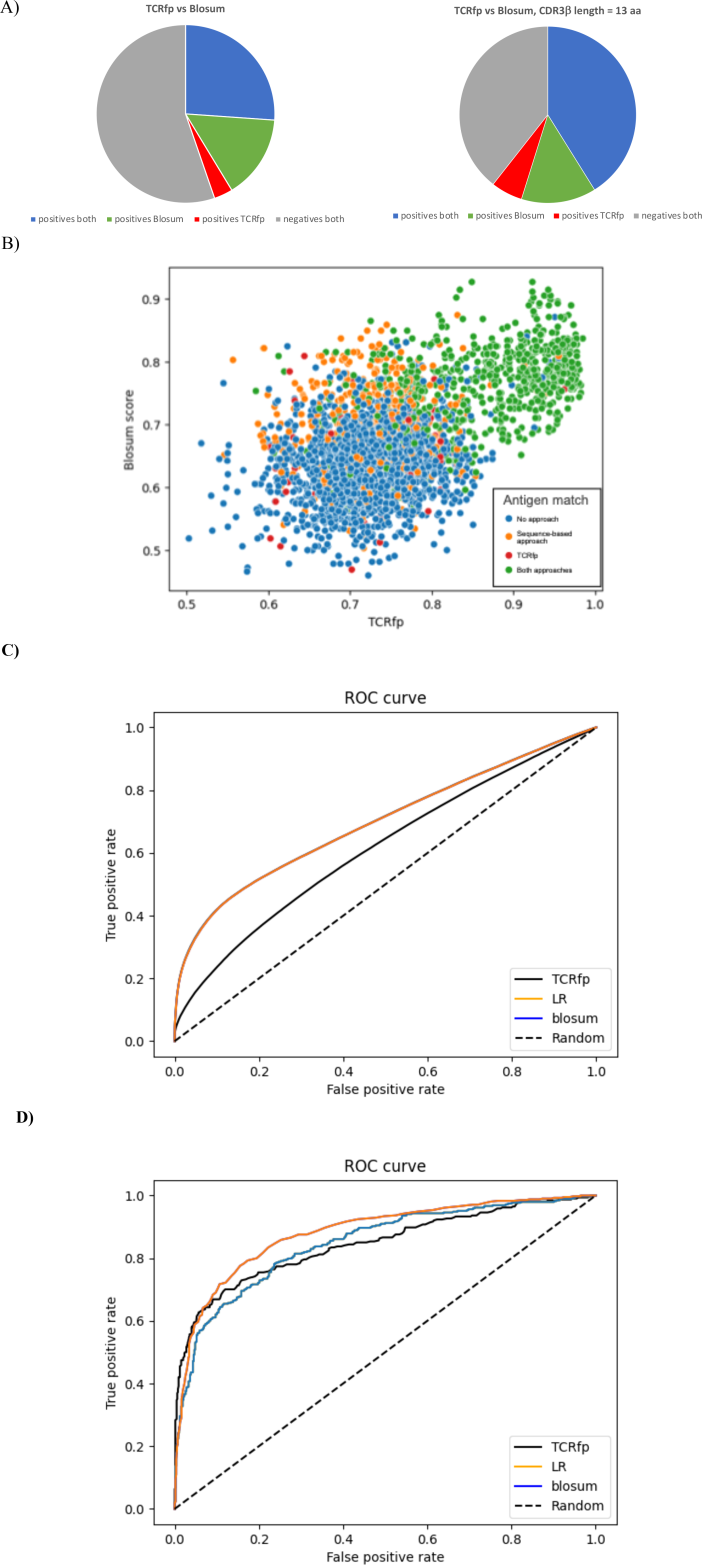
**(A)** Success of the sequence-based approach and TCRfp for pairing two TCRs of the same specificity at Rank 1. The % of TCRs successfully paired by both approaches in blue, only by the sequence-based approach in green and only by TCRfp in red. The % of TCRs not paired, whatever the approach, is in grey. **(B)** Relationship between the TCRfp and the sequence-based obtained for each possible TCR, used as a reference, and its closest TCR according to each method, taken from the external validation set. The sequence-based score is normalized for an easier comparison. Each dot represents a TCR according to the similarity of its closest TCR obtained with the TCRfp placed in the X axis and the sequence similarity of its closest TCR according to the sequence-based score placed in the Y axis. The coloring system represents the capacity of an approach to predict if the closest TCR according to it is sharing the same specificity. Blue: no approach could predict correctly the correct specificity, Orange: only the sequence-based approach made the correct prediction, Red: only TCRfp made the correct prediction, Green: both approaches made the correct prediction. **(C)** Receiver Operating Characteristic curves (ROC) for the validation set of 3213 TCRs computed using TCRfp, the sequence-based approach (blosum) and a combination of both approaches via a logistic regression. All the possible TCR pairs distances considered 3213*3213. **(D)** Receiver Operating Characteristic curves (ROC) for the validation set of 3213 TCRs computed using TCRfp, the sequence-based approach (blosum) and a combination of both approaches via a logistic regression (LR). For each given TCR just the TCR with highest similarity was considered and therefore 3213 data points.

To further evaluate the performance of our structure-based approach, TCRfp, we compared it with a purely sequence-based method by applying a BLOSUM62 similarity score to our validation set. Briefly, we aligned the sequences of the 6 CDRs loops using pairwise alignment and calculated their similarity using the BLOSUM62 matrix. We used an open gap penalty of -3 and an extension gap penalty of -1 (43). Further details of this sequence-based approach are provided in the methods section. The comparison between these two methods is provided in **Table 1** and in **Figure 5**.

The sequence-based approach can match correctly TCRs binding identical pMHC in 41.4% of the cases, while the success rate of TCRfp approach is 29.8%. When the length of the CDR3β of the reference TCR is 13, both approaches work better altogether and individually, with 59.6% of the TCRs properly matched, irrespective to the threshold (**Figure 5A**). Importantly, we observed that 26.1% of the TCRs were correctly matched by both approaches, 15.2% only by the sequence-based approach and 3.4% exclusively by TCRfp.

We analyzed the cases where TCRfp accurately identified TCRs with the same specificity while the sequence-based approach did not. Clearly, the sequence-based method struggles when there are sequence discrepancies. For example, consider 10X ID 00018 (composed of the genes TRAV12-2, TRAJ45, TRBV28, TRBJ1-5, CDR3a: CAGGGGGADGLTF, and CDR3b: CASTLTGLGQPQHF). TCRfp paired it correctly at rank 1 with 10X ID 00017 (composed of the genes TRAV12-2, TRAJ42, TRBV28, TRBJ2-3, CDR3a: CAVTHYGGSQGNLI, and CDR3b: CASLRSAVWADTQYF), both binding the peptide ELAGIGILTV. The TCRfp similarity for this pair was 0.80, with a root mean square deviation (RMSD) of 0.76 Å for all heavy atoms. In contrast, the sequence-based approach scored this pair at only 0.56. It erroneously paired 10X ID 00018 at rank 1 with 10X ID 01495 (composed of the genes TRAV12-2, CDR3a: CAVISGGGADGLTF, TRAJ45, TRBV28, CDR3b: CASTIALGYEQYF), which binds the NLNCCSVPV peptide, with a similarity score of 0.70. TCRfp gave this pair a similarity score of 0.44 and did not predict shared specificity. Interestingly, the RMSD between their 3D structures is only 0.31 Å. Despite the higher sequence similarity and smaller RMSD between the last two TCRs, they did not cluster together using TCRfp, highlighting the method’s ability to prioritize shape and biophysical properties, which the sequence-based approach misses. Thus, TCRfp provides valuable insights in cases where the sequence-based approach fails.

**Figure 5B**, shows TCRs taken from the validation set, scored according to the TCRfp and sequence-based values of the closest TCRs in the dataset according to each approach used separately. The color coding indicates whether the closest TCR according to each approach is sharing the same specificity. We observed that the majority of the TCRs correctly matched by both approaches are within the area comprised above a 0.8 TCRfp score and a 0.7 sequence-based score. Interestingly, some TCR that show a relative low sequence-based score are correctly paired using TCRfp, as it can be seen below the 0.6 threshold of the sequence-based score. This highlights again that our new TCRfp approach can correctly predict the specificity in some cases where the sequence-based approach would fail. All the data used to construct the **Figure 5** can be found in the **Supplementary Table 8**.

The 30 most frequent peptides in the validation set are described and their respective frequency in the validation set is also presented in the **Supplementary Table 9**. We observe that GILGFVFTL is the most frequent peptide in the validation set, representing 14.6% of the TCRs. This is also largely the most frequent peptide in the subset of TCRs correctly predicted by both approaches, with 34.6% of the TCRs correctly paired by both approaches recognizing this peptide. Interestingly, we observe that TCRs recognizing RLRAEAQVK are never correctly paired when using the sequence-based score indicating that shape has, more than a sequence, an effect in the recognition of the peptide (data shown in the **Supplementary Table 9**). Interesting too is the fact that TCRs recognizing AVFDRKSDAK were never correctly paired by both approaches at the same time and are more frequent in the subset of TCRs correctly paired by the TCRfp (11.9% for TCRfp-uniq and 3.7% for SeqBased-uniq). The comparisons presented in the **Figure 5B** allowed us to understand peptides where shape-based approaches can be extremely relevant to find the TCR specificity.

On this validation set, the sequence-based approach generally outperformed TCRfp (**Table 1**, **Figure 5**). As anticipated based on the best predictive performance across different ranks (**Table 1**), the sequence-based method achieved a high Area Under the Receiver Operating Curve (AUC) of 0.70, while TCRfp scored 0.61 (**Figure 5C**). This may indicate that the biophysical and structural characteristics of the TCR contribute less to the prediction than the sequence. Nevertheless, TCRfp performed better than the sequence-based score in particular cases as discussed upwards and, as for example, for pairing TCRs that recognize the RLRAEAQVK peptide (data shown in the **Supplementary Table 9**). To leverage the strengths of both methods, we combined the sequence-based score with TCRfp via a logistic regression (LR, see methods section 3.8). This combination resulted in an AUC of 0.70 and an example of its application on a private test set is shown in the next section, highlighting an increase predicting specificities. As previously mentioned, our analysis include predictive ability based on ranks and thresholds, as this closely mirrors the approach we plan to use in real-world applications (**Table 1**). For instance, when assessing the specificity of an orphan TCR, we will compare it against a list of TCRs with known specificity, identifying the closest matches. Only the most similar (top-ranked) TCRs with high similarity will be considered. We believe that **Table 1** provides a clearer description of this process than the ROC curves. Concomitantly, if instead of using all the 3213*3213 TCR pairs we only use for each given TCR, the TCR excluding itself with highest similarity, corresponding to rank 1 and therefore 3213 data points, we obtain an AUC of 0.84 for TCRfp and 0.85 for blosum and 0.87 for LR (**Figure 5D**).

### Example of TCRfp application

To assess the usefulness of TCRfp in processing clinical data, we applied it to a private set of 45 TCRs. This ensured a fair comparison, as this set of TCRs with known specificities was not used by us or by our competitors to develop the approaches. On top of comparing TCRfp with a purely sequence-based approach and with a logistic regression that combines TCRfp and the sequence-based we have also compared with the gold standard approaches TCRpcDist-3D (30), TCRdist3 (44) and TCRbase (web server: https://services.healthtech.dtu.dk/services/TCRbase-1.0/).

The results can be seen in the **Table 2**.

**Table 2:** Comparison of the success rate in paring a TCR with another one showing the same specificity, using different approaches applied to a private set of TCRs.

Rank	Method					
	TCRfp	Sequence- based	LR	TCRpcDist-3D	TCRdist3	TCRbase
Rank 1	22.2	46.7	48.9	44.4	46.7	44.4
Rank 2	35.6	60.0	62.2	64.4	62.2	46.7
Rank 5	57.8	75.6	73.3	75.6	71.1	–

We compared the TCRfp approach with the sequence-based approach, the logistic regression (LR) that combines both of them and TCRpcDist-3D, TCRdist3 and TCRbase. This comparison was done for the different ranks 1, 2 and 5.

**Figure 6:**
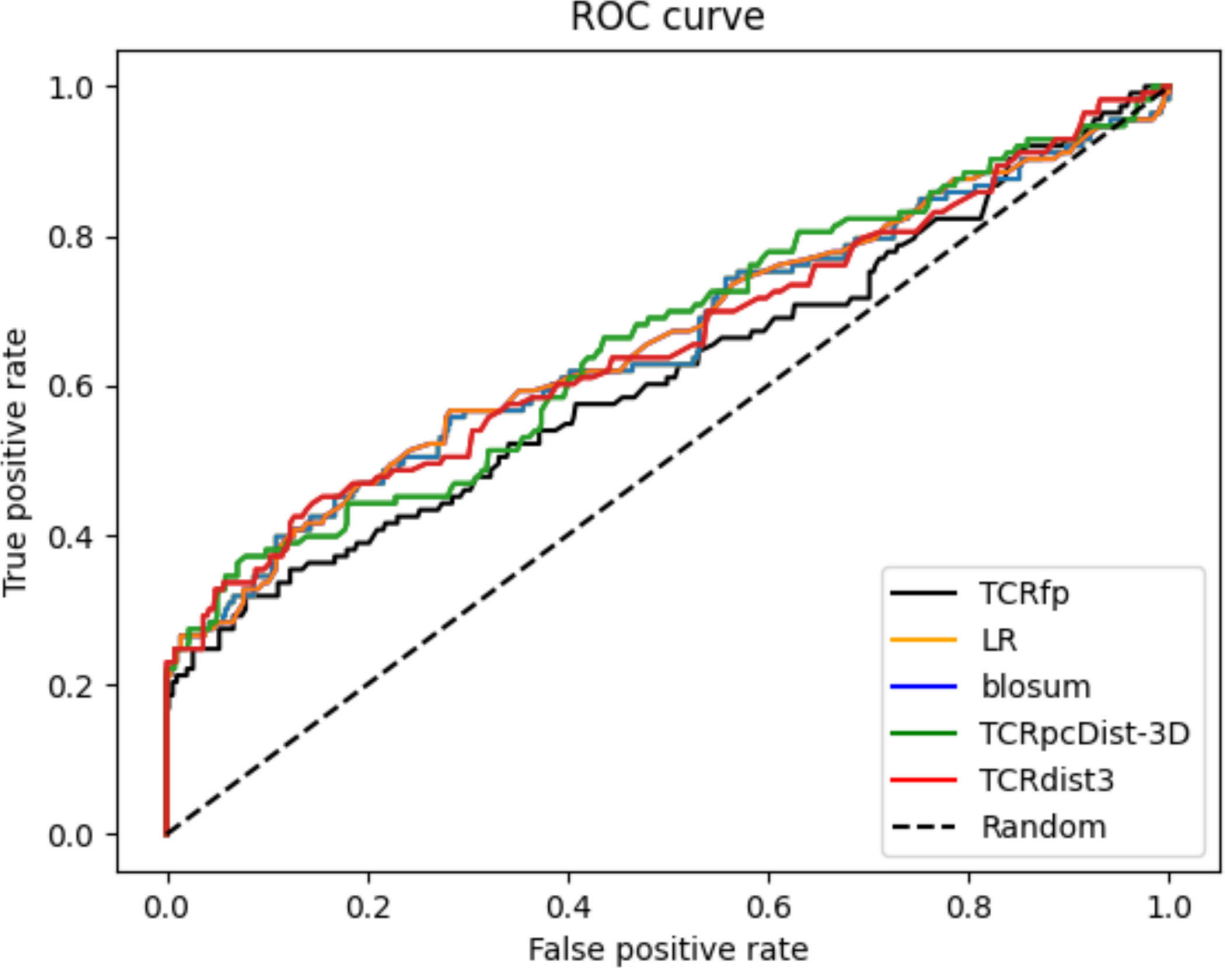
Receiver Operating Characteristic curves (ROC) for the private set 45 TCRs computed with the TCRfp approach the sequence-based approach, the logistic regression (LR) that combines both of them and the state-of-the art competitors TCRpcDist-3D and TCRdist3.

When tested on a private dataset (not included in any of the training of the used approaches) and integrated with a basic sequence-based method via logistic regression, TCRfp outperformed the competitors in predicting TCR specificity at rank 1. TCRfp, however, alone was shown as the least performant approach. ROC curves are also provided for all the approaches (**Figure 6**) except for TCRbase as we could not calculate all the 45*45 TCR distances (see Methods). The average AUC values were calculated after 5 cross-validations randomly taking 70% of the private TCRs as a test set and 30% as validation set. The average AUC values in % are for TCRfp, Blosum, LR, TCRpcDist-3D and TCRdist3 respectively 57+/-3, 65+/-2, 65+/-2, 67+/-1, 66+/-2. The classification reports with precision, recall and f1 score in **Supporting Information**.

### AI-based modeling does not enhance the TCRfp predictive ability

We compared the predictive performance of TCRfp pipeline using TCRmodel as a structural modelling engine, with an alternative TCRfp pipeline using an AI-based modelling approach. For the AI-based modelling approach, we used ImmuneBuilder (TCRBuilder2). The seminal paper on ImmuneBuilder shows that it can produce TCR structures with accuracy comparable to AlphaFold-Multimer, while being over a hundred times faster and without needing large sequence databases or multiple sequence alignments (51). We were unable to model 510 TCRs from the validation set using TCRBuilder2, which is why we are discussing the results for a set of 2703 TCRs in **Table 3**. See **Supporting Information** labeled **DataSheet 1** for details about the structures that were not modelled. When comparing the set of 2703 TCRs, we found that, although we cannot be certain of the exact structure due to the absence of experimental data, the RMSD for each loop averaged within 2Å when comparing TCRmodel within our pipeline and TCRBuilder2, suggesting that the models from both are not particularly different.

**Table 3:** Comparing the predictive ability of TCRfp pipeline using TCRmodel versus using TCRBuilder2. .

TCRfp variant	Threshold	Rank 1	Correctly paired TCRs	Rank 2	Correctly paired TCRs	Rank 5	Correctly paired TCRs	Total TCRs	%TCRs clustered
TCRfp – TCRmodel in FP pipeline	No threshold	32.3	872	37.4	1012	45.7	1235	2703	100%
TCRfp – AI modeling	No threshold	29.9	809	34.9	944	44.5	1203	2703	100%

When compared the predictive ability of TCRfp pipeline using TCRmodel versus using the AI-based approach, **Table 3**, we observe the following:

- For the validation set of 2’703 TCRs (out of the original 3’213), the TCRfp pipeline using TCRmodel identified a TCR pair sharing the same specificity at rank 1 in 32.3% of cases, while the AI-based TCRfp pipeline found such a pair at rank 1 in 29.9% of cases.
- Looking at the top 2 ranked TCRs, the TCRfp pipeline using TCRmodel identified a TCR sharing the same specificity in 45.7% of cases, while the AI-based pipeline yielded a slightly lower value of 44.5%.

When it comes to TCR specificity prediction, the TCRmodel approach appears to be more effective for this set of structures. We note that the TCRmodel pipeline involves multiple attempts and stringent distance filters, which were developed in-house to improve accuracy. We believe the Electroshape method compresses 3D information to such a degree that further advancements in modeling may not substantially enhance the overall predictions. Nonetheless, we will continue to monitor developments in AI technologies and reassess their potential integration into the TCRfp pipeline.

## Discussion

This work introduced a new structural approach for TCR pairing and clustering based on ES5D fingerprints, called TCRfp, which involves the encoding of a 3D structure into a 1D vector. Although this unique approach was initially developed for small molecules, we hereby successfully applied the ES5D FPs on a substantial set of highly diverse TCRs. This novel approach has demonstrated the possibility of rapidly pairing TCRs in a way that correlates with their antigen specificity, underlining the importance of TCRs structural features for understanding their binding properties beyond purely sequence information. Importantly, while TCRfp proved competitive, the present study represents only a first exploration of the potential of structure-based TCR fingerprinting approaches in this context. These approaches offer many possibilities of improvement and adaptation.

Here, we applied the TCRmodel approach to model thousands of TCR sequences obtained from 10xGenomics (52). By subjecting the approach to a larger number of sequences and a more diverse range of TCRs than in the original publication, we observed that modeling certain TCRs could prove challenging. This difficulty arises from the high flexibility of the CDR loops coupled with the limited number of templates available for reference. To improve modelling accuracy, we have developed a pipeline that is able to improve the quality of the TCR models and discard the bad models. We successfully modelled 82% of the class I TCR repertoire in 10xGenomics which is already a substantial TCR coverage for applications like vaccination and immunotherapy, where the goal is to pinpoint some strong candidates and not to comprehensively study the entire repertoire.

We also explored the possibility to provide a single generalized definition of the FP to improve the efficiency of the calculations using genetic algorithms. Genetic algorithms were applied over different training sets, using a similarity-based score (GA MATCH) or a distance-based score (GA MaxD). For both methods, the parameters were modified and tuned over different trials resulting in a better overall score and predictive ability for TCRfp optimized by the MATCH objective function, although it was built using a smaller training set, which could have led to overfitting. Of note, the approach using the original definition of the TCRfp based on centroids positioned on the tip of the loop, performed better than the GA-improved centroid definitions. Clearly, adapting the position of the centroid to each TCR proved more accurate to cluster TCRs in a way it that matches their specificity.

We also demonstrated that TCRfp is able to correctly pair TCRs which are not correctly matched with a sequence-based approach. The fact that our approach can predict TCR specificity when the sequence information is not sufficient, underlines the importance of the structure and biophyshicochemical properties of TCR loops for the TCR-pMHC interaction and TCR specificity determination.

We additionally explored the ability of combining TCRfp with a sequence-based score using logistic regression. We found that the combined approach increased the accuracy of TCR specificity prediction. The logistic regression weights give a higher contribution to the sequence based term, in line with the fact that the biophysical and structural characteristics of the TCRs contribute less to the prediction than the sequence. Still, the enhanced predictive power of the combined approach is in line with the importance of incorporating TCR structural parameters, as well as charges and lipophilicity information, in peptide specificity prediction. When applying the approach to an experimental dataset not used for the training, we observed an improvement in the performance of the logistic regression compared over the sequence-only approach for rank1 and rank2. We thus demonstrated the ability of TCRfp to complement a sequence-based approach and provides additional meaningful information not encoded in sequence-based algorithms. While a combined approach using TCRfp and a pure sequence-based method shows potential, it is still in an early stage and requires further investigation. Therefore, we are providing access to TCRfp as a standalone tool. This allows users to analyze TCR distances based on the biophysicochemical properties of the 3D structure and to integrate TCRfp with the sequence-based methods of their choice as needed.

Finally, switching from TCRmodel to the AI modeling approach TCRBuilder2 did not improve the predictive ability of TCRfp. We believe that the Electroshape method compresses 3D data to such an extent that further improvements in modeling may not lead to significant gains in overall predictions. However, we will continue to monitor advancements in AI technologies and reassess their potential integration into the TCRfp pipeline. Additionally, incorporating a faster approach would certainly be beneficial for the TCRfp approach.

This work demonstrates the feasibility of rapid structure-based approach for TCR repertoire analysis, TCR clustering and potentially TCR specificity prediction, with possible clinical applications. TCRfp thus introduces a new class of approaches for TCR pairing and clustering that can shed some light on the complex structural mechanism underlying TCR-pMHC recognition.

## Data Availability

TCRfp is a pipeline piloting different codes available as a free and login free webservice beta.swiss tcr.ch, allowing to use it command-line. To calculate TCR similarities using TCRfp please follow the instructions in the README-TCRfp-webservice.” Instructions on how to use the TCRfp web service can be found in the **Supporting Information** in the file README-TCRpcDist-webservice. An input file example is also provided. All data are available in the article/**Supplementary Material**. Any additional information is available from the correponding author upon request.

## References

[1] Mukhopadhyay M . Diving into the TCR repertoire. Nat Methods. (2021) 18:30. doi: 10.1038/s41592-020-01031-0, PMID: 33408389

[2] Hudson D Fernandes RA Basham M Ogg G Koohy H . Can we predict T cell specificity with digital biology and machine learning? Nat Rev Immunol. (2023) p:1–11. doi: 10.1038/s41577-023-00835-3, PMID: 36755161 PMC9908307

[3] Vujovic M Degn KF Marin FI Schaap-Johansen AL Chain B Andresen TL . T cell receptor sequence clustering and antigen specificity. Comput Struct Biotechnol J. (2020) 18:2166–73. doi: 10.1016/j.csbj.2020.06.041, PMID: 32952933 PMC7473833

[4] Heumos L Schaar AC Lance C Litinetskaya A Drost F Zappia L . Best practices for single-cell analysis across modalities. Nat Rev Genet. (2023) 24:550–72. doi: 10.1038/s41576-023-00586-w, PMID: 37002403 PMC10066026

[5] Deering RP Blumenberg L Li L Dhanik A Jeong S Pourpe S . Rapid TCR: Epitope Ranker (RAPTER): a primary human T cell reactivity screening assay pairing epitope and TCR at single cell resolution. Sci Rep. (2023) 13:8452. doi: 10.1038/s41598-023-35710-7, PMID: 37231180 PMC10212918

[6] Schmidt J Chiffelle J Perez MAS Magnin M Bobisse S Arnaud M . Neoantigen-specific CD8 T cells with high structural avidity preferentially reside in and eliminate tumors. Nat Commun. (2023) 14:3188. doi: 10.1038/s41467-023-38946-z, PMID: 37280206 PMC10244384

[7] Wang L Lan X . Rapid screening of TCR-pMHC interactions by the YAMTAD system. Cell Discov. (2022) 8:30. doi: 10.1038/s41421-022-00386-2, PMID: 35379810 PMC8979966

[8] Leinonen R Sugawara H Shumway M Collaboration INSD . The sequence read archive. Nucleic Acids Res. (2011) 39:D19–21. doi: 10.1093/nar/gkq1019, PMID: 21062823 PMC3013647

[9] Gowthaman R Pierce BG . TCR3d: The T cell receptor structural repertoire database. Bioinformatics. (2019) 35:5323–5. doi: 10.1093/bioinformatics/btz517, PMID: 31240309 PMC6954642

[10] Data, i . Available online at: https://clients.adaptivebiotech.com/immuneaccess (Accessed September 9, 2019).

[11] Biotechnologies, A . TCR-Antigen Map (2020). Available online at: https://www.adaptivebiotech.com/tcr-antigen-map/ (Accessed September 9, 2019).

[12] Glanville J Huang H Nau A Hatton O Wagar LE Rubelt F . Identifying specificity groups in the T cell receptor repertoire. Nature. (2017) 547:94–8. doi: 10.1038/nature22976, PMID: 28636589 PMC5794212

[13] Nielsen M Lundegaard C Worning P Lauemøller SL Lamberth K Buus S . Reliable prediction of T-cell epitopes using neural networks with novel sequence representations. Protein Sci. (2003) 12:1007–17. doi: 10.1110/ps.0239403, PMID: 12717023 PMC2323871

[14] Katayama Y Yokota R Akiyama T Kobayashi TJ . Machine learning approaches to TCR repertoire analysis. Front Immunol. (2022) 13:858057. doi: 10.3389/fimmu.2022.858057, PMID: 35911778 PMC9334875

[15] Montemurro A Schuster V Povlsen HR Bentzen AK Jurtz V Chronister WD . NetTCR-2.0 enables accurate prediction of TCR-peptide binding by using paired TCRα and β sequence data. Commun Biol. (2021) 4:1060. doi: 10.1038/s42003-021-02610-3, PMID: 34508155 PMC8433451

[16] Zoete V Irving M Ferber M Cuendet MA Michielin O . Structure-based, rational design of T cell receptors. Front Immunol. (2013) 4:268. doi: 10.3389/fimmu.2013.00268, PMID: 24062738 PMC3770923

[17] Schmidt J Smith AR Magnin M Racle J Devlin JR Bobisse S . Prediction of neo-epitope immunogenicity reveals TCR recognition determinants and provides insight into immunoediting. Cell Rep Med. (2021) 2:100194. doi: 10.1016/j.xcrm.2021.100194, PMID: 33665637 PMC7897774

[18] Ehrlich R Kamga L Gil A Luzuriaga K Selin LK Ghersi D . SwarmTCR: a computational approach to predict the specificity of T cell receptors. BMC Bioinf. (2021) 22:422. doi: 10.1186/s12859-021-04335-w, PMID: 34493215 PMC8422754

[19] Lin X George JT Schafer NP Chau KN Birnbaum ME Clementi C . Rapid assessment of T-cell receptor specificity of the immune repertoire. Nat Comput Sci. (2021) 1:362–73. doi: 10.1038/s43588-021-00076-1, PMID: 36090450 PMC9455901

[20] Lanzarotti E Marcatili P Nielsen M . Identification of the cognate peptide-MHC target of T cell receptors using molecular modeling and force field scoring. Mol Immunol. (2018) 94:91–7. doi: 10.1016/j.molimm.2017.12.019, PMID: 29288899 PMC5800965

[21] Consortium P . Protein Data Bank: the single global archive for 3D macromolecular structure data. Nucleic Acids Res. (2019) 47:D520–8. doi: 10.1093/nar/gky949, PMID: 30357364 PMC6324056

[22] Gowthaman R Pierce BG . TCRmodel: high resolution modeling of T cell receptors from sequence. Nucleic Acids Res. (2018) 46:W396–401. doi: 10.1093/nar/gky432, PMID: 29790966 PMC6030954

[23] Yin R Ribeiro-Filho HV Lin V Gowthaman R Cheung M Pierce BG . TCRmodel2: high-resolution modeling of T cell receptor recognition using deep learning. Nucleic Acids Res. (2023) 51:W569–76. doi: 10.1093/nar/gkad356, PMID: 37140040 PMC10320165

[24] David A Islam S Tankhilevich E Sternberg MJE . The alphaFold database of protein structures: A biologist’s guide. J Mol Biol. (2022) 434:167336. doi: 10.1016/j.jmb.2021.167336, PMID: 34757056 PMC8783046

[25] Klausen MS Anderson MV Jespersen MC Nielsen M Marcatili P . LYRA, a webserver for lymphocyte receptor structural modeling. Nucleic Acids Res. (2015) 43:W349–55. doi: 10.1093/nar/gkv535, PMID: 26007650 PMC4489227

[26] Armstrong MS Finn PW Morris GM Richards WG . Improving the accuracy of ultrafast ligand-based screening: incorporating lipophilicity into ElectroShape as an extra dimension. J Comput Aided Mol Des. (2011) 25:785–90. doi: 10.1007/s10822-011-9463-8, PMID: 21822723

[27] Armstrong MS Morris GM Finn PW Sharma R Moretti L Cooper RI . ElectroShape: fast molecular similarity calculations incorporating shape, chirality and electrostatics. J Comput Aided Mol Des. (2010) 24:789–801. doi: 10.1007/s10822-010-9374-0, PMID: 20614163

[28] Ostmeyer J Christley S Toby IT Cowell LG . Biophysicochemical motifs in T-cell receptor sequences distinguish repertoires from tumor-infiltrating lymphocyte and adjacent healthy tissue. Cancer Res. (2019) 79:1671–80. doi: 10.1158/0008-5472.CAN-18-2292, PMID: 30622114 PMC6445742

[29] Moris P De Pauw J Postovskaya A Gielis S De Neuter N Bittremieux W . Current challenges for unseen-epitope TCR interaction prediction and a new perspective derived from image classification. Brief Bioinform. (2021) 22(4). doi: 10.1093/bib/bbaa318, PMID: 33346826 PMC8294552

[30] Perez MAS Chiffelle J Bobisse S Mayol-Rullan F Bugnon M Bragina ME . Predicting antigen-specificities of orphan T cell receptors from cancer patients with TCRpcDist. Adv Sci (Weinh). (2024) 11:e2405949. doi: 10.1002/advs.202405949, PMID: 39159239 PMC11516110

[31] Berman HM Westbrook J Feng Z Gilliland G Bhat TN Weissig H . The protein data bank. Nucleic Acids Res. (2000) 28:235–42. doi: 10.1093/nar/28.1.235, PMID: 10592235 PMC102472

[32] Bagaev DV Vroomans RMA Samir J Stervbo U Rius C Dolton G . VDJdb in 2019: database extension, new analysis infrastructure and a T-cell receptor motif compendium. Nucleic Acids Res. (2020) 48:D1057–62. doi: 10.1093/nar/gkz874, PMID: 31588507 PMC6943061

[33] Meng EC Pettersen EF Couch GS Huang CC Ferrin TE . Tools for integrated sequence-structure analysis with UCSF Chimera. BMC Bioinf. (2006) 7:339. doi: 10.1186/1471-2105-7-339, PMID: 16836757 PMC1570152

[34] Stein A Kortemme T . Improvements to robotics-inspired conformational sampling in rosetta. PloS One. (2013) 8:e63090. doi: 10.1371/journal.pone.0063090, PMID: 23704889 PMC3660577

[35] Leaver-Fay A Tyka M Lewis SM Lange OF Thompson J Jacak R . ROSETTA3: an object-oriented software suite for the simulation and design of macromolecules. Methods Enzymol. (2011) 487:545–74. doi: 10.1016/B978-0-12-381270-4.00019-6, PMID: 21187238 PMC4083816

[36] Alford RF Leaver-Fay A Jeliazkov JR O'Meara MJ DiMaio FP Park H . The rosetta all-atom energy function for macromolecular modeling and design. J Chem Theory Comput. (2017) 13:3031–48. doi: 10.1021/acs.jctc.7b00125, PMID: 28430426 PMC5717763

[37] Lefranc MP Pommié C Ruiz M Giudicelli V Foulquier E Truong L . IMGT unique numbering for immunoglobulin and T cell receptor variable domains and Ig superfamily V-like domains. Dev Comp Immunol. (2003) 27:55–77. doi: 10.1016/S0145-305X(02)00039-3, PMID: 12477501

[38] Dunbar J Deane CM . ANARCI: antigen receptor numbering and receptor classification. Bioinformatics. (2016) 32:298–300. doi: 10.1093/bioinformatics/btv552, PMID: 26424857 PMC4708101

[39] Lefranc MP . Immunoglobulin and T cell receptor genes: IMGT(^®^) and the birth and rise of immunoinformatics. Front Immunol. (2014) 5:22. doi: 10.3389/fimmu.2014.00022, PMID: 24600447 PMC3913909

[40] Pettersen EF Goddard TD Huang CC Couch GS Greenblatt DM Meng EC . UCSF Chimera–a visualization system for exploratory research and analysis. J Comput Chem. (2004) 25:1605–12. doi: 10.1002/jcc.20084, PMID: 15264254

[41] Hinz T Weidmann E Kabelitz D . Dual TCR-expressing T lymphocytes in health and disease. Int Arch Allergy Immunol. (2001) 125:16–20. doi: 10.1159/000053792, PMID: 11385284

[42] Daina A Michielin O Zoete V . SwissADME: a free web tool to evaluate pharmacokinetics, drug-likeness and medicinal chemistry friendliness of small molecules. Sci Rep. (2017) 7:42717. doi: 10.1038/srep42717, PMID: 28256516 PMC5335600

[43] Henikoff S Henikoff JG . Amino acid substitution matrices from protein blocks. Proc Natl Acad Sci U.S.A. (1992) 89:10915–9. doi: 10.1073/pnas.89.22.10915, PMID: 1438297 PMC50453

[44] Mayer-Blackwell K Fiore-Gartland A Thomas PG . Flexible distance-based TCR analysis in python with tcrdist3. Methods Mol Biol. (2022) 2574:309–66. doi: 10.1007/978-1-0716-2712-9_16, PMID: 36087210 PMC9719034

[45] Bradley P . Structure-based prediction of T cell receptor:peptide-MHC interactions. Elife. (2023) 12. doi: 10.7554/eLife.82813, PMID: 36661395 PMC9859041

[46] Martin YC Kofron JL Traphagen LM . Do structurally similar molecules have similar biological activity? J Med Chem. (2002) 45:4350–8. doi: 10.1021/jm020155c, PMID: 12213076

[47] Burley SK Berman HM Kleywegt GJ Markley JL Nakamura H Velankar S . Protein data bank (PDB): the single global macromolecular structure archive. Methods Mol Biol. (2017) 1607:627–41. doi: 10.1007/978-1-4939-7000-1_26, PMID: 28573592 PMC5823500

[48] Daina A Michielin O Zoete V . SwissTargetPrediction: updated data and new features for efficient prediction of protein targets of small molecules. Nucleic Acids Res. (2019) 47:W357–64. doi: 10.1093/nar/gkz382, PMID: 31106366 PMC6602486

[49] Zoete V Daina A Bovigny C Michielin O . SwissSimilarity: A web tool for low to ultra high throughput ligand-based virtual screening. J Chem Inf Model. (2016) 56:1399–404. doi: 10.1021/acs.jcim.6b00174, PMID: 27391578

[50] Huang H Wang C Rubelt F Scriba TJ Davis MM . Analyzing the Mycobacterium tuberculosis immune response by T-cell receptor clustering with GLIPH2 and genome-wide antigen screening. Nat Biotechnol. (2020) 38:1194–202. doi: 10.1038/s41587-020-0505-4, PMID: 32341563 PMC7541396

[51] Abanades B Wong WK Boyles F Georges G Bujotzek A Deane CM . ImmuneBuilder: Deep-Learning models for predicting the structures of immune proteins. Commun Biol. (2023) 6:575. doi: 10.1038/s42003-023-04927-7, PMID: 37248282 PMC10227038

[52] 10xGenomics . 10xGenomics . Available online at: https://www.10xgenomics.com/ (Accessed September 9, 2019).

