# Supplementary material for: A fingerprint approach to pioneer structure-based T cell receptor repertoire analysis and specificity prediction
Source: Front Immunol. 2025 Nov 7;16:1688805. doi: 10.3389/fimmu.2025.1688805 (PMC12634567; doi:10.3389/fimmu.2025.1688805)
Supplement: Supplementary file 1 [file DataSheet1.docx]

Supporting Information

**AI based modelling within TCRfp**

**We compared the predictive performance of our TCRfp pipeline using TCRmodel with an alternative TCRfp pipeline using an AI-based modeling approach.**

For the AI-based model, we used ImmuneBuilder (TCRBuilder2), as we have been utilizing these tools in the lab for other projects, making them easier to integrate. Furthermore, the seminal paper on ImmuneBuilder shows that it can produce TCR structures with accuracy comparable to AlphaFold-Multimer, while being over a hundred times faster and without needing large sequence databases or multiple sequence alignments ([https://www.nature.com/articles/s42003-023-04927-7](https://www.nature.com/articles/s42003-023-04927-7)).

When compared the predictive ability of TCRfp pipeline using TCRmodel versus using the AI-based approach, **table 1** below and table 3 in the manuscript, we observed the following:

- For the validation set of 2703 TCRs (out of the original 3213), the TCRfp identified a TCR pair sharing the same specificity at rank 1 in 32.3% of the cases using the TCRmodel version and in 29.9% of the cases using the AI-based version.
- Looking at the top 5 ranked TCRs, the TCRfp using TCRmodel identified a TCR sharing the same specificity in 45.7% of cases, while the AI-based version yielded a slightly lower value of 44.5%.

**Table 1** . Comparing the predictive predictive ability of TCRfp pipeline using TCRmodel versus using the AI-based approach TCRBuilder2.

|  | Threshold | Rank1 | Correctly Paired TCRs | Rank2 | Correctly Paired TCRs | Rank5 | Correctly Paired TCRs | Total TCRs | %TCRs  clustered |
| --- | --- | --- | --- | --- | --- | --- | --- | --- | --- |
| TCRfp – **TCRmodel in FP pipeline** | No threshold | 32.3 | 872 | 37.4 | 1012 | 45.7 | 1235 | 2703 | 100% |
| TCRfp – AI modeling | No threshold | 29.9 | 809 | 34.9 | 944 | 44.5 | 1203 | 2703 | 100% |

We note that we were unable to model 510 TCRs from the validation set using TCRBuilder2, which is why we are discussing the results for a set of 2703 TCRs that could be modeled by both approaches.

Regarding the structures that could not be modeled with TCRBuilder2, as an example, we provide a case where an alpha chain could not be modeled. The output message was: “Sequence provided as an A chain is not recognized as an A chain.” The issue seemed to arise because the amino acids in the C-terminal portion of the TRAV and the N-terminal portion of the TRAJ did not match the expected terminal amino acids of the CDR3alpha for this TCR. **Figure 1** below. This discrepancy may explain why TCRBuilder2 was unable to model it. However, the VDJ database score for this TCR is 2, indicating high confidence in the sequence. The original reference for this TCR is doi: 10.1002/1521-4141(200211)32:11<3181::AID-IMMU3181>3.0.CO;2-2. Although TCRBuilder2 could not model it, we consider a strenght the fact that TCRmodel was able to handle this case. Still the important here is the comparison of the approach with an AI-based version and we did it successfully for 2703 TCRs.


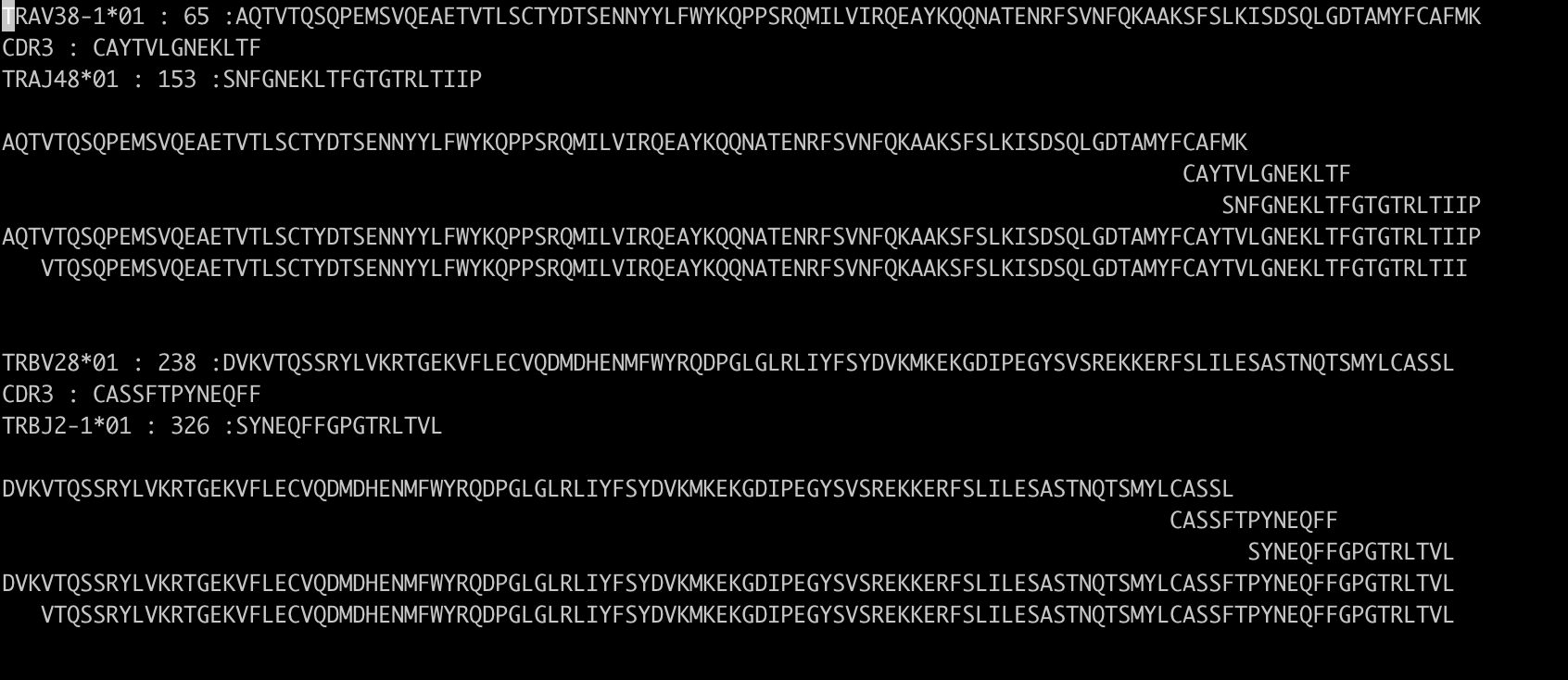


**Figure 1**. Example of a TCR from the validation set that could be modeled with TCRmodel and could not be modelled with ImmuneBuilder.

The TCRmodel approach within TCRfp incorporates multiple attempts and stringent distance filters (as detailed in the results in the paper), which may explain some of the higher accuracy observed in certain models for this set of 2703 TCRs. We provide a comparison of the models from TCRBuilder2 and TCRmodel below. When comparing the set of 2703 TCRs, we found that, although we cannot be certain of the exact structure due to the absence of experimental data, the root mean square deviation (RMSD) for each loop averaged within 2Å when comparing TCRmodel within our pipeline and TCRBuilder2, suggesting that the models from both are not particularly different**. Figure2,** **figure 3** and **table 2**.


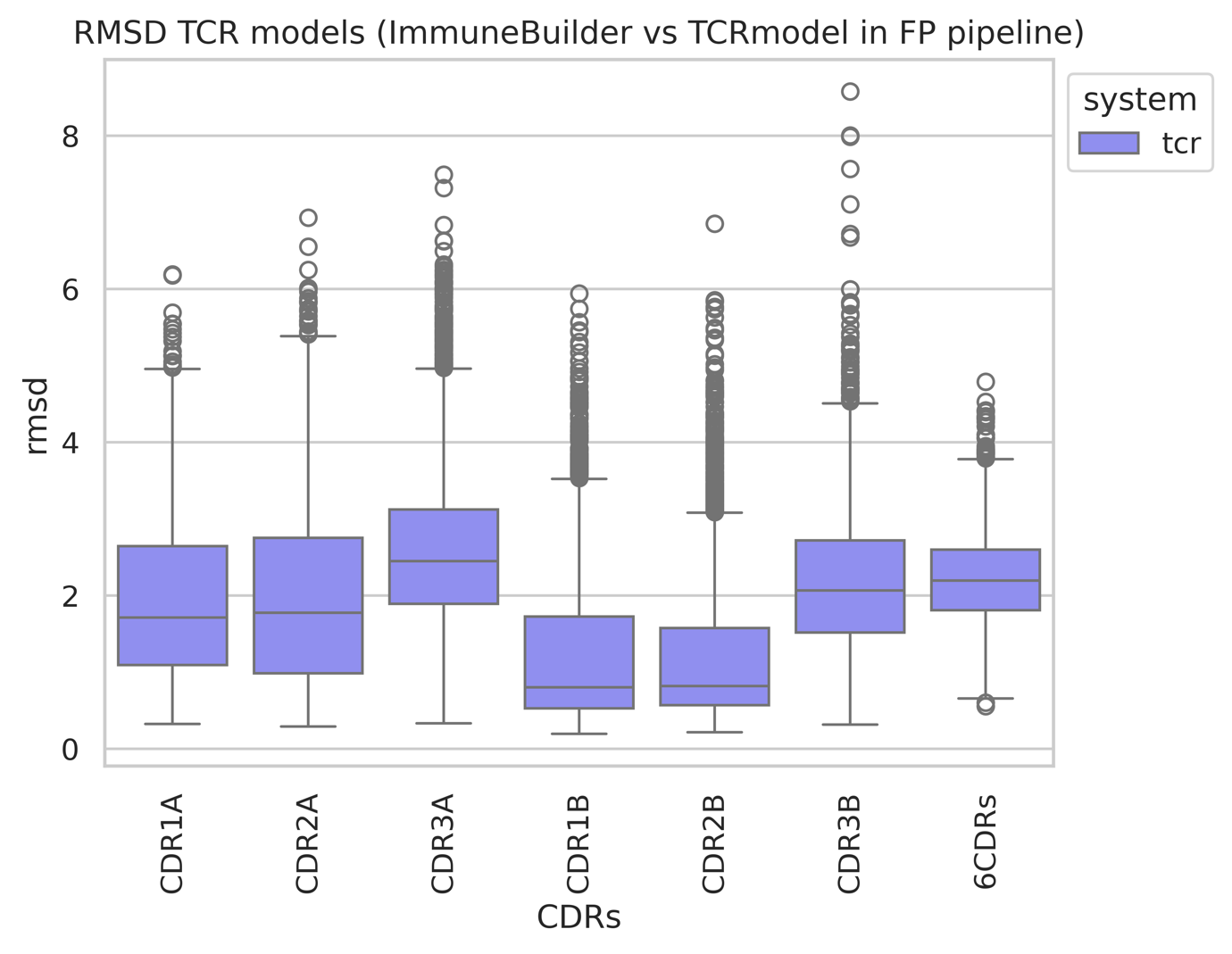


**Figure 2**. Boxplot for the RMSD between the models in TCRmodel as within our pipeline and the models in ImmuneBuilder(TCRBuilder2) for each of the six CDRs individually and all the 6 CDRs together. The validation set of 2703 TCRs considered. RMSDs are given in Angstroms (Å).


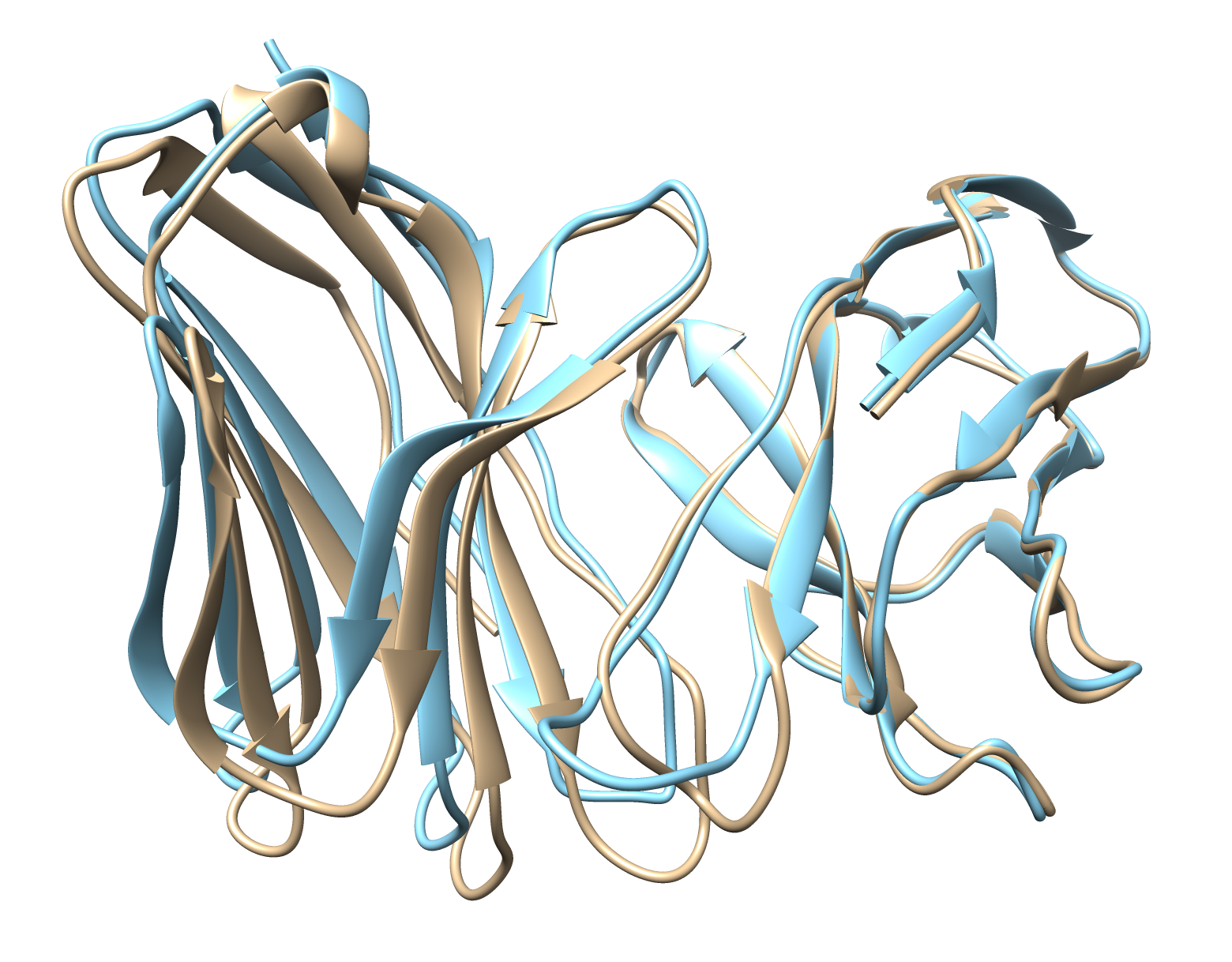


**Figure 3**. TCR models comparison. In brown TCR model created with TCRmodel and in blue created with ImmuneBuilder (TCRBuilder2). The TCR genes and CDRs are TRAV12-2, TRAJ45, CDR3A: CAVGGGADGLTF,TRBV6-5,TRBJ2-7,CDR3B: CASSYSATGGEQYF and specificity to ELAGIGILTV .The average root mean square deviation for the 6 CDRs is 1.8 Angstrom considering uniquely main chain atoms, N,C,O and Calpha.

**Table 2**. The mean, standard deviation, maximum , minimum and quartiles between the models in TCRmodel as within our pipeline and the models generated by ImmuneBuilder for each of the six CDRs individually and all the 6 CDRs together. The validation set of 2703 TCRs considered. RMSDs are given in Angstroms (Å).

| \|  \| CDR1a \| CDR2a \| CDR3a \| CDR1b \| CDR2b \| CDR3b \| 6CDRs \| \| --- \| --- \| --- \| --- \| --- \| --- \| --- \| --- \| \| mean \| 1.94 \| 1.99 \| 2.61 \| 1.23 \| 1.24 \| 2.20 \| 2.24 \| \| std \| 1.07 \| 1.22 \| 1.07 \| 0.98 \| 1.01 \| 0.95 \| 0.60 \| \| min \| 0.33 \| 0.29 \| 0.33 \| 0.20 \| 0.22 \| 0.32 \| 0.56 \| \| 0.25 \| 1.10 \| 0.99 \| 1.89 \| 0.53 \| 0.57 \| 1.52 \| 1.81 \| \| 0.50 \| 1.71 \| 1.78 \| 2.45 \| 0.80 \| 0.82 \| 2.07 \| 2.20 \| \| 0.75 \| 2.64 \| 2.75 \| 3.13 \| 1.73 \| 1.58 \| 2.72 \| 2.60 \| \| max \| 6.19 \| 6.93 \| 7.50 \| 5.94 \| 6.85 \| 8.58 \| 4.79 \| |
| --- | --- | --- | --- | --- | --- | --- | --- | --- | --- | --- | --- | --- | --- | --- | --- | --- | --- | --- | --- | --- | --- | --- | --- | --- | --- | --- | --- | --- | --- | --- | --- | --- | --- | --- | --- | --- | --- | --- | --- | --- | --- | --- | --- | --- | --- | --- | --- | --- | --- | --- | --- | --- | --- | --- | --- | --- | --- | --- | --- | --- | --- | --- | --- | --- |

We believe the Electroshape method compresses 3D information to such a degree that further advancements in modeling may not substantially enhance the overall predictions. Nonetheless, we will continue to monitor developments in AI technologies and reassess their potential integration into the TCRfp pipeline.
