# Supplementary material for: A fingerprint approach to pioneer structure-based T cell receptor repertoire analysis and specificity prediction
Source: Front Immunol. 2025 Nov 7;16:1688805. doi: 10.3389/fimmu.2025.1688805 (PMC12634567; doi:10.3389/fimmu.2025.1688805)
Supplement: Supplementary file 2 [file DataSheet2.docx]

**SI – More on the Heuristic Searches Explorations**

The MATCH objective function consists in optimising the measure of similarity between a training set of TCRs binding the same pMHC. **Supplementary Table 6** provides a detailed summary of the parameters used during the MATCH explorations and the specific values regarding diversity and variability of the GA exploration are described in the **Supplementary Table 7.**

Our hypothesis was that the MATCH objective function would eventually drive the centroids towards the CDRs or at the vicinity of the place where the TCR-pMHC interaction takes place, due to the importance of this region for the TCR specificity. As an illustration, **Figure 1A**, shows the 6 centroids obtained as best solutions of two different MATCH runs via random initial exploration, plotted in the cartesian space, along with a TCR model as a reference. While these positions are far from the tip of the loop and sometimes even from the TCR surface, interestingly, some centroids coordinates converge towards the same positions in different MATCH runs. The best overall solution obtained with MATCH is the one displayed on the left (in A, B, C) of **Figure 1A**, which provided a peptide identity score in the training set of 36.5% ± 5.0, significantly better than random, i.e. 7.7% ± 2.5 (p<0.0001). The *peptide identity* score measures, for each TCR in each set, how frequently the closest TCR according to TCRfp binds the same peptide. (**See Materials and Methods, subsection TCR similarity scoring)**. To assess the ability of the MATCH-optimized TCRfp to generate TCR clusters that correlate with their specificity, we built hierarchical trees for the training set. The nodes of the hierarchical tree were then coloured according to the pMHC the corresponding TCR can bind. As a measure of the quality of the clustering, we used a quantitative measure determined by the pMHC-distance. The pMHC-distance was defined as the average branch length distance between all possible pairs of TCR nodes that recognize the same pMHC. The ability of TCRfp MATCH to cluster 1 of the 10 sets of the training set is represented in a clustering tree in **Figure 2**. Our best MATCH-determined TCRfp version achieved 46% of *peptide identity* (with an average score of 36.5% ± 5.0 over the 10 sets used for the run). It also provides the lowest number of colour changes from node to node, with 83 changes compared to a mean of 88.6 ± 4.0 on the ten sets. This tree obtained a pMHC-distance a 0.79. The average pMHC-distance of this TCRfp version over all the ten sets of TCRs is 0.76 ± 0.03. We observed a large standard deviation of the *peptide identity* score (± 5.0) obtained by TCRfp on the ten TCR sets. This underlines a substantial dependency of TCRfp efficiency on the specific set of TCRs under investigation potentially due to the small size of TCR sets that are available – the latter itself a consequence of the fact that only a small number of pMHC have many TCRs known to bind to them – or both. Overall, the algorithm succeeded to cluster together TCRs based on their peptide recognition.

An alternative scoring function, MaxD, was introduced, attempting to improve the centroids. Contrarily to the MATCH function, which directly assesses if a TCR close to a reference TCR is likely to bind the same pMHC, the goal of the MaxD objective function is to separate all TCRs as much as possible, hoping that the TCRs with closest distances will spontaneously correspond the TCRs binding the same antigens. One advantage of this approach is that it does not require equal size sets of TCR with known specificities, to explore the best centroids. All available TCRs were consequently used, whatever their specificities. Thus, we could constitute a much larger training set of 2831 TCRs. These TCRs can be seen in **Supplementary Table 1**. In these new runs, the MaxD scoring function is thus the average distance between all possible TCRfp pairs in the training set and the objective is to converge to centroids that maximize as much as possible this value. The details of the MaxD optimizations are specified in the **Supplementary Tables 6 and 7**.

**Figure 1. A) Comparison of the centroids obtained with the two best solutions from the heuristic searches using the MATCH objective function, starting from a random initial position. For each panel.** The values for the charge range from -1 (in red) to 1 (in blue) and the diameter of each centroid correlates with the normalized charge and lipophilicity. See the scales in the bottom left of the last subfigure 3. The centroids of the best solution are coloured in green and the centroids of the second-best solution in orange. Different views of the two solution solutions represented with a TCR for reference: **1**. Front view of the TCR **2**. Side view rotated 90º to the right **3.** Side view rotated 90º to the left. The reference TCR used for the visualization is a model obtained with our pipeline corresponding to the ID 46 (encoded by the TRAV8-4, TRAJ20, TRBV19 and TRBJ1-1 genes, CDR3α: CAVSPNDYKLSF and CDR3b: CASSIRSTTEAFF, binding the peptide GILGFVFTL. **B) Best 2 solutions obtained with the TCRfp MATCH GA-based algorithm.** The solutions correspond to the FP values which are organized according to the weighted parameters (WP) for the charge and lipophilicity, respectively, and the 6 centroids (C1, C2, C3, C4, C5, C6). The numbers given to each centroid are just a reference, as they all originate from randomized values they hold no representation of their position in space for the GA-based algorithms, unlike the tip-of-the-loop original TCRfp.

**Figure 2. TCR hierarchical tree built with ES5D FPs for the TCRfp MATCH approach applied to a set of 117 TCRs.** Importantly, the tree was obtained using only the ES5D-based similarities between TCRs, without any information regarding the pMHC they bind. It is only after the creation of this tree that the TCRs were coloured according to the pMHC they recognise. The sequence of the latter is also noted in the tree. The tree was obtained from the highest-ranked set of the best-ranked TCRfp parameters determined using the MATCH objective function, with a 45.0% *peptide identity* *score* (average score of the 10 sets according to the *peptide identity score:* 36.5% ± 5.0). The values obtained with the pMHC distance, and the node colour changes are 0.79 and 83, respectively.

**
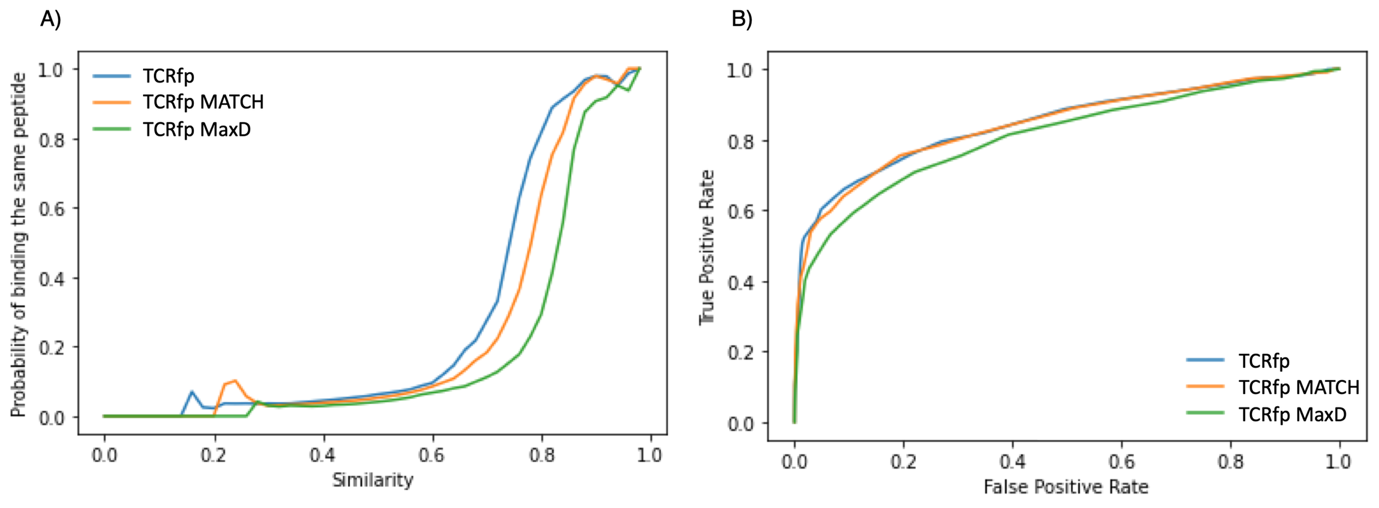
**

**Figure 3. A) Relationship between TCR similarity (as calculated for an external validation set) and the probability of binding the same peptide.** Comparison between the centroids placed in the tip of the loop approach (tip-of-the-loop TCRfp) and the best centroids position for the heuristic search (MATCH-optimized TCRfp) and the best centroids position obtained with the distance-based scoring (MaxD-optimized TCRfp). B) Relationship between the True Positive Rate and the False Positive Rate obtained with the three different approaches. The three approaches are much better than random.

When applied to an external validation set of 3’213 TCRs, the definition of TCRfp, with centroids positioned on the tips of the loop, TCRfp^TOL^ provided an averaged *peptide identity score* of 29.8% (Rank 1; no threshold, p-value <0.0001 when compared with random). The best solutions of the heuristic searches, MATCH and MaxD, TCRfp^MATCH^ and TCRfp^MaxD^ runs led to averaged *peptide identity scores* of 28.2% and 29.6%, respectively (Rank 1; no threshold, p-value <0.0001 when compared with random 4.8% Rank 1; no threshold, p-value <0.0001). To assess the ability of our approach to pair TCRs with the same specificity at different FP similarity values, we calculated how frequently the TCR with closest distance (Rank 1) the two closest TCRs (Rank 2) and the 5 closest TCRs (Rank 5) share the same specificity (predictive ability). (positive pairs) vs how often these pairs do not share the same specificity (negative pairs). The distribution of these frequencies can be seen in the **Figure 3** for the different centroid definitions. Again, TCRfp^TOL^ provides the best predictive ability. The threshold TCR similarity values, above which the frequency of positive pairs is higher than the frequency of negatives ones increases in this order: TCRfp^TOL^ - Rank 5; 0.74, Rank 2: 0.77; Rank 1: 0.85, TCRfp^MATCH^: Rank 5: 0.78; Rank 2: 0.82; Rank 1: 0.86, TCRfp^MaxD^: Rank 5: 0.82; Rank 2: 0.86; Rank 1: 0.88. These findings suggest we can be more accurate by using similarity thresholds. When considering a similarity threshold of 0.8 and rank5, TCRfp^TOL^ achieves a predictive ability of 75.48%, substantially better than TCRfp^MATCH^ (54.1%) and TCRfp^MaxD^ (36.5%).

**Figure 3.** A) Distributions of the frequency with which the closest TCR (Rank1; subfigures 1, 4, 7), at least one of the two closest (Rank2; subfigures 2, 5, 8) or among the 5 closest TCRs (Rank 5; 3, 6, 9) to a reference TCR binds the same pMHC as a function of the similarity calculated with TCRfp for the three different approaches: TCRfp^TOL^ (subfigures 1, 2 and 3), TCRfp^MATCH^ (subfigures 4, 5 and 6) or TCRfp^MaxD^ (subfigures 7, 8 and 9).

To better understand the impact of the different approaches on the mean and standard deviation as well as the minimum and maximum of the 18 values of the FP vectors, the latter were calculated for the entire validation set. The average standard deviation of the 18 values of the TCRfp^TOL^ vector is higher (4.31) compared to the heuristic searches (TCRfp^MATCH^: 1.18 and TCRfp^MaxD^: 2.40), showing that each fingerprint vector entry was more variable in the TCRfp approach. **See Table 1**. These results allow us to conclude that using the TCRfp^TOL^ approach provided more variability to the FP entry values, thus, making them more distinguishable. In addition, the preliminary definition of the centroids has the advantage of being independent from the position and the orientation of the molecule in space, making the ES5D vectors themselves independent from this, i.e. there is no need to align the TCR on the cartesian centre and axes before calculating its FP. Also, it may indicate that an extensive tunning of the parameters translates into a potential overfitting of the algorithm, thus, the reduced variability and efficacy of the FPs generated using the parameters optimized by heuristic search approaches.

Defining the TCRfp using the tip of the loop of each TCR as the centroid outperforms the use of generalized centroids, as attempted in the heuristic searches. We observe that adapting the centroid position for each TCR as in the TCRfp definition using the tip of the loop improves clustering accuracy, bringing it into closer alignment with their specificity. From now on, when mentioned TCRfp we are discussing TCR using the tip of the loop as centroids definitions.

**Table 1.**

**A) Comparison of the mean and standard deviation of the FP vectors obtained from the TCRs of the external validation set with the three different approaches.** The three approaches being compared are: TCRfp preliminary, TCRfp from MATCH runs and TCRfp from MaxD runs. Each vector is constructed with 18 values coming from three values per each centroid according to the ES5D protocol described in Methods. In the table we can see the mean obtained for each value for all the TCRs from the set according to each approach as well as the standard deviation given inside the brackets. **B) Comparison of the minimum and maximum values of the FP vectors obtained from the TCRs of the external validation set with the three different approaches.** The three approaches being compared are: TCRfp preliminary, TCRfp from MATCH runs and TCRfp from MaxD runs. Each vector is constructed with 18 values coming from three values per each centroid according to the ES5D protocol described in Methods. In the table we can see the minimum and maximum numbers obtained for each value for all the TCRs from the set according to each approach
