# Supplementary material for: A fingerprint approach to pioneer structure-based T cell receptor repertoire analysis and specificity prediction
Source: Front Immunol. 2025 Nov 7;16:1688805. doi: 10.3389/fimmu.2025.1688805 (PMC12634567; doi:10.3389/fimmu.2025.1688805)
Supplement: Supplementary file 3 [file DataSheet3.docx]

Supplementary Materials for

**A Fingerprint Approach to Pioneer Structure-Based T Cell Receptor Repertoire Analysis and Specificity Prediction**

Francesca Mayol-Rullan^1,2^, Marine Bugnon^1,2^, Marta A. S. Perez^1,2^ and Vincent Zoete^1,2,3^

*Corresponding author. Email: vincent.zoete@unil.ch

^1^Computer-aided Molecular Engineering Group, Department of Fundamental Oncology,
Lausanne University, Ludwig Institute for Cancer Research, Route de la Corniche 9A, 1066 Epalinges, Switzerland.

^2^Molecular Modelling Group, SIB Swiss Institute of Bioinformatics,
Quartier UNIL-Sorge, Bâtiment Génopode, 1015 Lausanne, Switzerland.

^3^Department of Oncology UNIL-CHUV, University of Lausanne, Ludwig Institute of

**Table S1.**

Structural information of the PDB TCR HLA-A2 class I structures used to calculate the preliminary results obtained from the Protein Data Bank (PDB). The columns contain the following information, from left to right: PDB ID, α chain, β chain and their cognate peptide (aligned among the rest of peptides). **TableS1.xlsx**

Table S2.

List of all the IDs for the set of 92 experimental structures of TCR class I employed to calculate the distances for the distance-based filter. **TableS2.csv**

1ao7, 1bd2, 1mi5, 1oga, 1qrn, 1qse, 1qsf, 2ak4, 2bnq, 2bnr, 2esv, 2f53, 2gj6, 2nx5, 2p5e, 2pye, 2vlj, 2vlk, 2vlr, 2ypl, 3d39, 3d3v, 3dxa, 3ffc, 3gsn, 3h9s, 3kpr, 3kps, 3kxf, 3mv7, 3mv8, 3mv9, 3pwp, 3qdg, 3qdj, 3qdm, 3qeq, 3qfj, 3sjv, 3uts, 3utt, 3vxr, 3vxs, 4eup, 4ftv, 4g8g, 4g9f, 4jfe, 4jrx, 4jry, 413e, 4prh, 4pri, 4prp, 4qrp, 5c07, 5c08, 5c09, 5c0a, 5c0b, 5c0c, 5d2l, 5d2n, 5e6i, 5e9d, 5eu6, 5eu0, 5hhm, 5hho, 5hyj, 5isz, 5jhd, 5jzi, 5men, 5nht, 5nme, 5nmf, 5nmg, 5nqk, 5nme, 5nmf, 5nmg, 5nqk, 5tez, 5w1v, 5w1w, 5wkf, 5wkh, 6am5, 6amu, 6avf, 6avg, 6bj2

Table S3.

List of all the modeled TCRs obtained from the 10xGenomics data base used in this project (10X id). The table contains information regarding all the genes and peptide specificity (pMHC = pHLA-A2) related to each TCR. The table also indicates if a 3D model was successfully obtained (tcr_model = Yes) and if that TCR was included within the TCRfp MATCH (match_set = Yes) or TCRfp MaxD (maxd_set = Yes) training sets. **TableS3.csv**

**Table S4.**

TCRs from the VDJ database used as a validation set. The table contains the information regarding all the genes and peptide specificity. **TableS4.csv**

**Table S5.**

List of the modeled private collection of 45 CD8+ TCR sequences with known pMHC from 4 melanoma cancer patients (Mel #1, Mel #2, Mel #3, Mel #4). The table contains the information regarding all the genes and peptide specificity. **TableS5.csv**

**Table S6.**

Summary of the GA best algorithms. The table gives information regarding the top 5 centroids scoring the higher peptide identity or distance, according to the respective algorithm used. The explorations were done using 10 training sets comprising an equal number of TCRs binding the same peptide. Each training set of the MATCH algorithms was composed of groups of 9 TCRs each binding the same antigen, among 13 different ones, generating a total of 117 TCRs per set whereas the MaxD algorithms were tested in a single test set of 3213 TCRs (specified in the ‘Test set’ column). For two specific trials we tested the algorithm including the entire length of the loop, as seen with the ‘entire loop’ label. The table also distinguishes between the scoring system used in each algorithm in the column ‘Heuristic search’, where we separate between MATCH or MaxD. In the column ‘Initial generation’ we can find if the initial generation was totally random or close to the tip of the loop (TOL). The ‘Maximum score’ column represents the highest value achieved by the training set, indicating the peptide identity and the distance for the MATCH and MaxD algorithms respectively; the ‘number of runs’ indicate the genetic algorithm repetitions that each algorithm underwent and the ‘mean final score’ shows the average of the training set for all the runs from the previous column. More specifically for the top 5 centroids, we can find the run identifier, their final score (peptide identity for MATCH and distance for MaxD), final C and P (charge and logP weighted parameters) as well as the rest of the fingerprint value (columns: ‘Run’, ‘Score’, ‘C’, ‘P’, ‘Centroids’ respectively). **TableS6.csv**

**Table S7.**

Detailed GA parameters which led to the best genomes among all those tested for the TCRfp MATCH and TCRfp MaxD approaches. The explorations were done using 10 training sets comprising an equal number of TCRs binding the same peptide. Each training set of the MATCH algorithms was composed of groups of 9 TCRs each binding the same antigen, among 13 different ones, generating a total of 117 TCRs per set whereas the MaxD algorithms were tested in a single test set of 3213 TCRs. Main parameters: number of centroids and dimensions explored in each algorithm, initial size of the population and number of offspring genomes generated during each generation and the number of parents used from the main population to perform the next generation. Parameters of the initial generation: range of values for the C and P weighting parameters and X, Y, Z, c and p centroid coordinates, from within the initial genome parameters could be selected to generate a random initial population. Reproduction parameters: number of mutations that could be used when generating each child genome (the value is randomly chosen each time), the range value from which were selected the random value that would be subtracted or added to the parameter to be mutated depending if the parameter is the weighted C, weighted P, the X,Y,Z coordinates or the C and P values, the crossover type used for the algorithm (if a single centroid was swapped or multiple centroids) and the crossover point (if the entire centroid was swapped or a random portion of it). **TableS7.csv**

**Table S8.**

TCR scores comparisons between the FP approach and the sequence-based approach. The table contains all the 10X ID (tcr_id) TCRs used in for the validation set, their peptide specificity (peptide), if the closest TCR obtained with the FP score (fp_MATCH = Yes) and the sequence-based score (BLOSUM_MATCH = Yes) are binding the same peptide, the respective antigens binding the closest TCRs (fp_peptide for the FP score peptide and BLOSUM_peptide for the sequence-based approach) and FP similarity (fp_score) and sequence similarity (BLOSUM_score) and the category according to the each approach MATCH. **Table S8.**

**Table S9.**

**Representation of each peptide in the set of TCRs with specificity correctly predicted by: both approaches (column 3), uniquely by sequence-based (SeqBased-uniq, column 4) and uniquely by TCRfp (TCRfp-uniq, column 5).** The representation is calculated as the ratio between the number of TCRs correctly paired binding a given peptide and the total number of TCRs correctly paired, at rank 1. The 30 most frequent peptides in the validation set are described and their respective frequency in the validation set is presented in column 2.

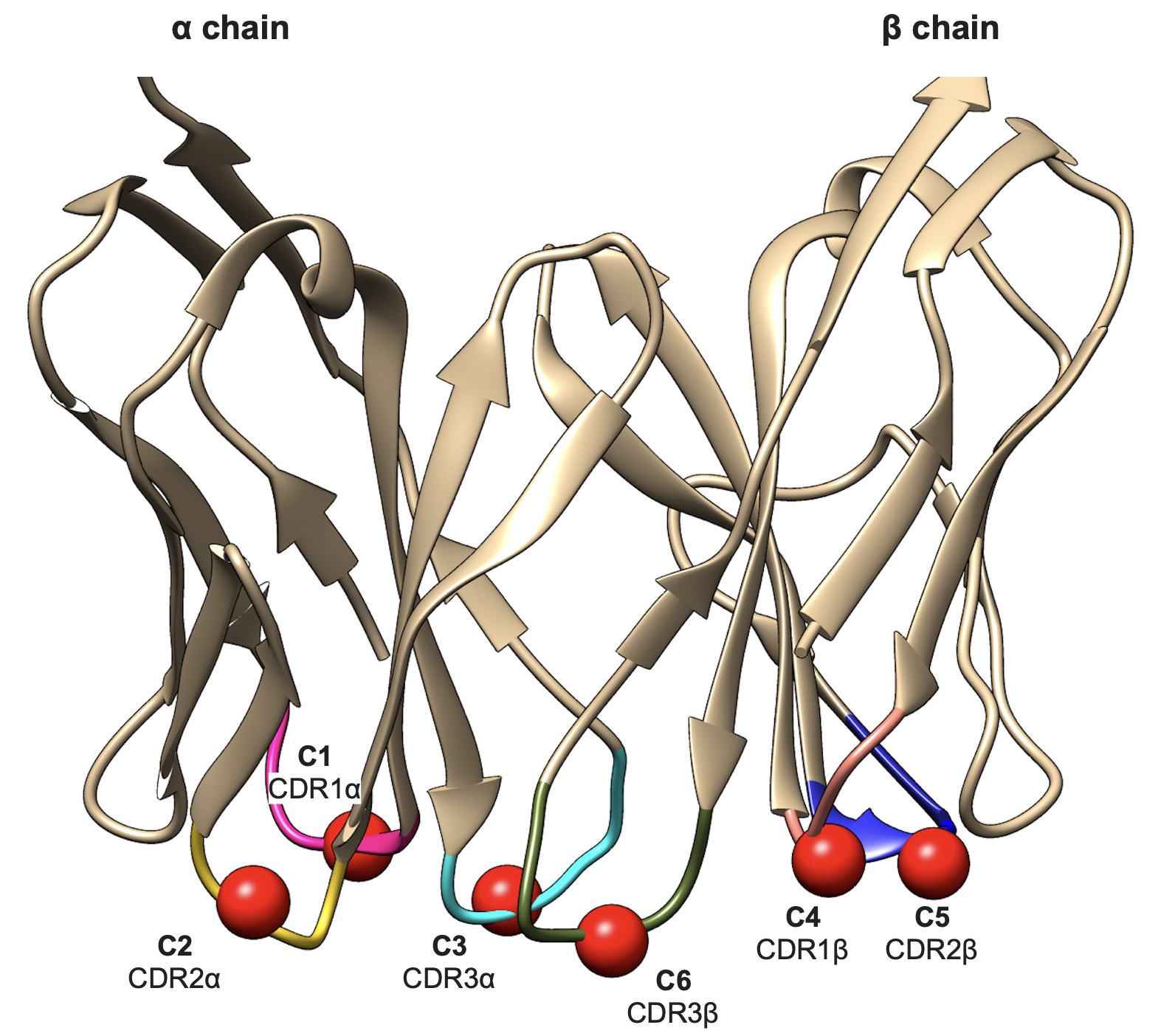


**Figure S1.**

**Positions of the 6 centroids (C1, C2, C3, C4, C5 and C6) in the TCR structure/model according to the first definition of the ES5D FP.** Each loop is coloured differently and highlighted with a red sphere that represents the carbon α of the middle residue and therefore the centroid. CDR1α is coloured in magenta, CDR2α in yellow, CDR3α in cyan, CDR1β in light red, CDR2β in dark blue and CDR3β in green.
