# Supplementary material for: A fingerprint approach to pioneer structure-based T cell receptor repertoire analysis and specificity prediction
Source: Front Immunol. 2025 Nov 7;16:1688805. doi: 10.3389/fimmu.2025.1688805 (PMC12634567; doi:10.3389/fimmu.2025.1688805)
Supplement: Supplementary file 4 [file Presentation1.zip › webservice/README-TCRfp-webservice.docx]

**TCRfp – Temporary webservice**

# Checking that the server is up and running

Please type

curl -s beta.swiss-tcr.ch:9876

or

curl -s beta.swiss-tcr.ch:9876/

or

curl -s beta.swiss-tcr.ch:9876/hello

If the webser is up and running, you receive a "Hello World!" message.

> curl -s beta.swiss-tcr.ch:9876

Hello World!

> curl -s beta.swiss-tcr.ch:9876/

Hello World!

> curl -s beta.swiss-tcr.ch:9876/hello

Hello World!

**# TCRfp similarity distances between TCRs using shape**

To launch a TCR similarity measure (1-distance) using TCRfp, type

**curl -s -F "myCDRs3D=@input.txt" beta.swiss-tcr.ch:9876/calcdist3D**

**where**

**- input.txt is the file containing the TCR info, one TCR per line**

**An example input file is attached**

**The input file contains 8 columns space separated: TCR identifier, TRAV, CDR3a, TRAJ, TRBV, CDR3b, TRBV and peptide recognized (if it is a TCR with unknown specificity you may keep column 8 empty or filled with orphan or NA).**

In the attached example, input.txt, the rows are:

1 TRAV13-1 CAASDSSASKIIFG TRAJ3 TRBV9 CASSVGKETQYFG TRBJ2-5 FAFGEPREL

2 TRAV14/DV4 CAMREPYYNQGGKLIFG TRAJ23 TRBV4-3 CASSQDRLARDTQYFG TRBJ2-3 FAFGEPREL

3 TRAV26-2 CILRAVYVRFG TRAJ43 TRBV5-5 CASSWKGVYNQPQHFG TRBJ1-5 RRWDEKAVDKSK

**Please make sure that your input.txt file contains no empty lines and strictly follows the specified format above, including the format used for describing the genes.**

## Before launching the calculations, the server will make several checks:

### If, for some technical reason, it is impossible to write the file on the server, the server is sending an Error message:

> curl -s -F "myCDRs3D=@input.txt" beta.swiss-tcr.ch:9876/calcdist3D

Error: the input file could not be written on our file system. Please, contact the SwissTCR team.

in case of major problem with the machine, we get

> curl -s -F "myCDRs3D=@input.txt" beta.swiss-tcr.ch:9876/calcdist3D

internal server error

### if the input file was empty, the job will not be submitted:

> curl -s -F "myCDRs3D=@input.txt" beta.swiss-tcr.ch:9876/calcdist3D

## Successful submission

If no problem was found , the distance calculation is submitted to the queue of the server. Then, **the user is provided with a unique Session Number**.

This Session Number will allow the user to check what is the status of the calculation, and retrieve the results in case of successful calculation.

> curl -s -F "myCDRs3D=@input.txt" beta.swiss-tcr.ch:9876/calcdist3D

> 403049986

Here, 403049986 is the Session Number of the submitted screening.

# Checking the status of a screening

A user can check the status of a job using the Session Number he/she received upon submission.

If the calculation is pending in the queue, waiting for its turn, the user will be informed, and will be provided with the number of jobs that are waiting before it in the queue.

If the job is currently running, the user will be informed and the running time will be sent.

The user is also informed if the job is terminated

> curl -s “beta.swiss-tcr.ch:9876/checksession?sessionNumber=403049986”

Calculation is in the queue. Number of jobs before yours: 12

... and later ...

> curl -s “beta.swiss-tcr.ch:9876/checksession?sessionNumber=403049986”

Calculation is in the queue. Number of jobs before yours: 4

... and later ...

curl -s “beta.swiss-tcr.ch:9876/checksession?sessionNumber=403049986”

> Calculation currently running. Run time: 0:02

... and later ...

> curl -s “beta.swiss-tcr.ch:9876/checksession?sessionNumber=403049986”

Calculation is finished

On top of the above checking, the system also verifies if the Session Number is correct, and if a problem prevented submitting the calculation for an existing Session

Wrong session number:

curl -s “beta.swiss-tcr.ch:9876/checksession?sessionNumber=40304998”

Error. There is no trace of session 40304998. Is the session number correct?

No job submitted for an existing session:

> curl -s “beta.swiss-tcr.ch:9876/checksession?sessionNumber=519879130”

Error. No calculation was submitted to the queue for session 519879130. Please resubmit. If failure persists, please, contact the Swiss-TCR team:

[vincent.zoete@unil.ch](mailto:vincent.zoete@unil.ch) ; [marta.perez@sib.swiss](mailto:marta.perez@sib.swiss)

# Cancelling a screening

The user can cancel a run that is currently running or pending in the queue.

This command will remove the calculation from the queue of the server:

curl -s -F "myCDRs3D=@input.txt" beta.swiss-tcr.ch:9876/calcdist3D

174252494

... and later ...

> curl -s “beta.swiss-tcr.ch:9876/cancelsession?sessionNumber=174252494”

Calculation of session 174252494 was cancelled

Also for this command, the web server checks that the user provided a valid Session Number

> curl -s “beta.swiss-tcr.ch:9876/cancelsession?sessionNumber=17425249”

Error. There is no trace of session 17425249. Is the session number correct?

# Retrieving the results of a screening

When a user has checked that his/her job is finished (see above), it is possible to retrieve the results.

Here is a full sequence, (i) submitting a job, (ii) regularly checking if the job is finished and (iii) retrieving the results when it is the case:

>curl -s -F "myCDRs3D=@input.txt" “beta.swiss-tcr.ch:9876/calcdist3D”

186742962

... and later ...

>curl -s “beta.swiss-tcr.ch:9876/checksession?sessionNumber=186742962”

Calculation is in the queue. Number of jobs before yours: 2

... and later ...

>curl -s “beta.swiss-tcr.ch:9876/checksession?sessionNumber=186742962”

Calculation currently running. Run time: 0:01

... and later ...

> curl -s “beta.swiss-tcr.ch:9876/checksession?sessionNumber=186742962”

Calculation is finished

**> curl -s “beta.swiss-tcr.ch:9876/retrievesession?sessionNumber=186742962” > output.out**

**The TCR similarities are retrieved to an output**

If the user is too impatient, the system can remind him to wait until the job is finished, before retrieving the results

> curl -s “beta.swiss-tcr.ch:9876/checksession?sessionNumber=437240532”

Calculation is in the queue. Number of jobs before yours: 1

> curl -s “beta.swiss-tcr.ch:9876/retrievesession?sessionNumber=437240532”

Error. Calculation is not finished. Impossible to retrieve the results.

Again, the webserver checks the Session Number and the existence of a slurm job, and informs the user in case of problem

> curl -s “beta.swiss-tcr.ch:9876/retrievesession?sessionNumber=43724053”

Error. There is no trace of session 43724053. Is the session number correct?

Each TCR model requires approximately one minute to generate. The total runtime will vary depending on the number of available CPUs on the server and the queue of pending jobs ahead of yours.
