# Supplementary material for: A fingerprint approach to pioneer structure-based T cell receptor repertoire analysis and specificity prediction
Source: Front Immunol. 2025 Nov 7;16:1688805. doi: 10.3389/fimmu.2025.1688805 (PMC12634567; doi:10.3389/fimmu.2025.1688805)
Supplement: Supplementary file 11 [file Table7.pdf]

Genetic Algorithm Parameters		Main parameters					Parameters of the Initial Generation				Reproduction Parameters						
**Algorithm**		**Number of centroids**	**Dimensions**	**Initial size**	**Offspring size**	**Parent range**	**Range weighted C**	**Range weighted P**	**Range X,Y,Z coordinates**	**Range C,P values**	**Mutation number**	**Mutation range weighted C**	**Mutation range weighted P**	**Mutation range X,Y,Z coordinates**	**Mutation range C, P values**	**Crossover type**	**Crossover point**
Original scoring	TCRfp HS	6	5	400	200	200	0/50	0/20	-30/30	-1/1	1/2	-2/2	-2/2	-2/2	-2/2	Multiple centroid whole	Random number of centroids swap
	TCRfp MaxD	6	5	400	200	200	30/50	0/5	-30/30	-1/1	2/4	-1/1	-1/1	-1/1	-1/1	Multiple centroid whole	Random number of centroids with swap of the entire centroids
	Strict centroid movement	6	5	400	200	200	0/100	0/50	Inital close to the tip of the loop position with a range of -1/1	-30/30	1/2	-2/2	-2/2	-2/2	-2/2	Multiple centroid whole	Multiple whole centroid swap
	New centoid positions	6	5	400	200	200	0/100	0/50	3 centroids starting from CDR3alpha 3 centroids starting from CDR3beta	-30/30	1/2	-5/5	-5/5	-10/10	-5/5	Multiple centroid whole	Multiple whole centroid swap
	TCRfp HS looplength	6	5	400	200	200	0/100	0/50	Inital close to the tip - exact position	-30/30	1/2	-2/2	-2/2	-2/2	-2/2	Multiple centroid whole - box limitation	Multiple whole centroid swap - Mutations can't go further a 3D box
	TCRfp MaxD looplength	6	5	400	200	200	0/100	0/50	Inital close to the tip - exact position	-30/30	2/4	-1/1	-1/1	-1/1	-1/1	Multiple centroid whole	Random number of centroids with swap of the entire centroids
	Restricted space	6	5	400	200	200	0/100	0/50	Inital close to the tip - exact position	-30/30	1/2	-2/2	-2/2	-2/2	-2/2	Multiple centroid whole - box limitation	Multiple whole centroid swap - Mutations can't go further a 3D box
	TCRfp MaxD TOL	6	5	400	200	200	0/100	0/50	Inital close to the tip - exact position	-30/30	1/2	-10/10	-10/10	-20/20	-10/10	Single centroid whole	Single random centroid swap
	TCRfp MaxD TOL 2.0	6	5	400	200	200	0/100	0/50	Inital close to the tip - exact position	-1/1	1/2	-10/10	-10/10	-20/20	-10/10	Multiple centroid break	Random number of centroids swap with random break inside the centroid
New scoring	Rank 2	6	5	400	200	200	0/50	0/50	-30/30	-10/10	2/4	-2/2	-2/2	-2/2	-2/2	Multiple centroid break	Random number of centroids swap with random break inside the centroid
	Rank 5	6	5	400	200	200	0/50	0/50	-30/30	-10/10	2/4	-2/2	-2/2	-2/2	-2/2	Multiple centroid break	Random number of centroids swap with random break inside the centroid
	Rank 5 relaxed	6	5	400	200	200	0/50	0/50	-30/30	-10/10	2/4	-2/2	-2/2	-2/2	-2/2	Multiple centroid break	Random number of centroids swap with random break inside the centroid
